# Supplementary material for: How is cervical cancer screening information communicated in UK websites? Cross-sectional analysis of content and quantitative presentation formats
Source: BMJ Open. 2019 Oct 28;9(10):e029551. doi: 10.1136/bmjopen-2019-029551 (PMC6830680; doi:10.1136/bmjopen-2019-029551)
Supplement: Supplementary data [file bmjopen-2019-029551supp001.pdf]

Supplementary materials for BMJ Open

How is cervical screening communicated in UK websites? A cross-sectional analysis of content and quantitative presentation formats

Yasmina Okan,[1 *] Samuel G. Smith,[2] Wändi Bruine de Bruin,[1,3]

[1]Centre for Decision Research, Leeds University Business School, University of Leeds, Leeds, UK

[2] Leeds Institute of Health Sciences, University of Leeds, Leeds, UK

[3]Department of Engineering and Public Policy, Carnegie Mellon University, Pittsburgh, US

* Corresponding author at Centre for Decision Research, Leeds University Business School, University of Leeds, Charles Thackrah Building, Leeds, LS2 9LB, UK; E-mail: y.okan@leeds.ac.uk, Phone: +44 113 343 2622.

Table S1. Websites coded, date of access for analysis, and estimated visits.

Organization	Link	Date	Total monthly visits (million) [c]	% of UK visitors [c]
British Society for Colposcopy and Cervical Pathology	https://www.bsccp.org.uk/	28/04/2017	- [d]	- [d]
BootsWebMD [a]	https://www.webmd.boots.com/	09/01/2018	- [d]	- [d]
Bupa UK	https:/www.bupa.co.uk	27/04/2017	1.11	86.15%
Cancer Research UK	https://www.cancerresearchuk.org	27/04/2017	4.46	47.45%
Jo's Cervical Cancer Trust [a]	https://www.jostrust.org.uk	27/04/2017	0.21	68.64%
LGBT Foundation [b]	http://lgbt.foundation	27/04/2017	0.06	66.66%
MacMillan Cancer Support [a]	https://www.macmillan.org.uk	27/04/2017	1.13	65.03%
Marie Stopes UK [b]	https://www.mariestopes.org.uk	27/04/2017	0.06	46.77%
NHS Choices (England) [a]	http://www.nhs.uk	20/03/2017	33.53	71.60%
NHS Inform (Scotland) [a]	https://www.nhsinform.scot	20/03/2017	2.96	55.43%
Patient Info	https://patient.info	27/04/2017	4.06	47.05%
Public Health Agency Northern Ireland [a]	http://www.cancerscreening.hscni.net	26/04/2017	0.40 [e]	81.37% [e]
Public Health Wales [a]	http://www.cervicalscreeningwales.wales.nhs.uk	20/04/2017	1.07 [e]	87.68% [e]
Women's Health Concern	https://www.womens-health-concern.org	28/04/2017	0.12	19.11%

Note: [a] Electronic leaflets and/or factsheets for the general public were available to download via a link this website, and were thus considered part of this resource; [b] Only an electronic leaflet was available and thus the analysis focuses on this resource; [c] Estimated data obtained from SimilarWeb (www.similarweb.com), representing visits in June 2019. Estimates aggregate data across all website subdomains and are computed by extrapolation from a small panel of users, and therefore need to be interpreted with caution; [d] Estimates not available due to insufficient data; [e] Estimates based on the general domain ("hscni.net" and "wales.nhs.uk")

Table S2. Codes from Kolthoff et al.'s (2016) checklist, with examples of corresponding statements. Codes marked with an asterisk were added by the authors.

**Information item**	**Example statements**
**Screening benefits**	
Risk reduction of developing cervical cancer	Regular screening can cut the risk of getting cervical cancer by 75%.
	Cervical screening prevents 8 out of 10 cervical cancers from developing.
Risk reduction of death from cervical cancer	No statements found
Risk reduction of total mortality	No statements found
* Lives saved yearly	Cervical screening saves around 5,000 lives every year in the UK.
	Over 1000 lives are saved every year through regular screening.
* Fall in incidence since introduction of screening	The number of women who develop cervical cancer has halved since the 1980s due to most women regularly having cervical screening.
	Since the screening programme was introduced in the 1980s, the number of cervical cancer cases has decreased by about 7% each year.
**Screening risks**	
Overdiagnosis/ overtreatment	The test can pick up minor abnormalities in cervical cells which could clear up by themselves.
	Your screening test may show up mild cell changes in your cervix, which would never have gone on to become cancer or caused you any problems. If this happens, you may receive treatment that you don't really need.
Pain/discomfort related to the test	Cervical screening tests are not painful, although some women find the speculum uncomfortable.
	You might feel some discomfort or pain - try to relax by taking slow, deep breaths as it may hurt more if you are tense.
False negative results	No screening test is 100% accurate. Some tests will be falsely reassuring (so-called false negative results) - where the test is reported as normal but an abnormality is present.
	Regular screening detects up to 80% of cervical abnormalities.
Psychological distress due to abnormal results	A diagnosis of and treatment for cervical abnormalities can cause significant anxiety for many women.
	Many women worry when an abnormality is found.

False positive results	False positive results might wrongly show changes in your cervix.
	Very occasionally, screening can show you have abnormal cells when in fact there's no problem. This is called a false positive result.
Risks related to treatment	For a few women, the treatments may cause problems such as bleeding afterwards or a small increase in future pregnancies of having the baby early.
	Very rarely, the cervix can become tightly closed after treatment. This known as stenosis. It can make it harder for the sperm to enter the womb and so can affect your chances of becoming pregnant naturally.
**Screening results**	
Possibility of inadequate result	Sometimes test results are declared 'unsatisfactory' because the slide was poorly prepared or difficult to read. In such cases, the test needs to be repeated and the patient will be called back.
	An inadequate result does not mean your smear was abnormal but that it was unreadable. This means that there were not enough cells in the sample and happens in about 3 out of every 100 tests taken.
Possibility of abnormal result	Most abnormal results from screening tests show only very minor changes.
	About 1 in 20 women will have a smear test result which is abnormal.
Positive predictive value	No statements found
* Possibility of cancer diagnosis	A cervical screening test can very occasionally find early cervical cancer. Most women with an abnormal test result have early cell changes and not cancer.
	It is extremely rare for cancer to be diagnosed from a cervical screening test. Less than one in 1,000 women's test results show invasive cancer.
**Cervical cancer statistics**	
Survival from cervical cancer	Overall, almost 2 out of 3 women (63%) survive ten years or more, and more than 8 out of 10 women (83%) will live at least one year.
Lifetime risk of developing cervical cancer	No statements found
Lifetime risk of dying from cervical cancer	No statements found
* Cervical cancer incidence	Around 3,000 women are diagnosed with cancer of the cervix every year.
	Each year in Northern Ireland, about 103 women are diagnosed with cervical cancer.
* Cervical cancer mortality	Sadly around 900 women die of cervical cancer each year in England.
	In Northern Ireland, 20–30 women die each year from cervical cancer.

Table S3a. Information items about cervical screening benefits, risks, results, and cervical cancer statistics (Part 1)

	BSCCP	Boots WebMD	Bupa UK	Cancer Research UK	Jo's Cervical Cancer Trust	LGBT Foundation	MacMillan Cancer Support
**Screening benefits**							
Risk reduction of developing cervical cancer	-	-	-	RR	RR	RR	0
Risk reduction of death from cervical cancer	-	-	-	-	-	-	-
Risk reduction of total mortality	-	-	-	-	-	-	-
* Lives saved yearly	-	-	N	N	N	-	N
* Fall in incidence since the introduction of screening [a]	N	-	-	N	-	-	N
**Screening risks**							
Overdiagnosis/overtreatment	V	-	V	V	V, N	V	V
Pain/discomfort related to the test	-	0	0	0	V	V	V
False negative results	-	-	0	0	V, N	-	V
Psychological distress due to abnormal results	0	-	0	0	V	V	0
False positive results	V	-	V	V	-	-	V
Risks related to treatment	V	-	-	V	V	-	V
**Screening results**							
Possibility of inadequate result	0	V	V, N	V	V	0	V
Possibility of abnormal result	1X, N	0	1X	1X, N	V, 1X	V, 1X	V
Positive predictive value	-	-	-	-	-	-	-
* Possibility of cancer diagnosis	V, 1X, N	-	-	V	V, 1X	V	V
**Cervical cancer statistics**							
Survival from cervical cancer	-	N	-	-	-	-	-
Lifetime risk of developing cervical cancer	-	-	-	-	-	-	-
Lifetime risk of dying from cervical cancer	-	-	-	-	-	-	-
* Cervical cancer incidence	-	N	N	-	N	-	-
* Cervical cancer mortality	-	-	-	-	N	N	-
**Type of appeals**							
Participation	x	x	x	x	x	x	x
Informed decision making	-	-	x	-	x	-	-

Note: - = item not present; 0 = not quantified; V = verbal quantifier; RR = numerical relative risk reduction; 1X = numerical 1-in-X; N = numerical (other); G = graph; x = item present (coding format not applicable); * codes added by the authors; [a] includes estimates of incidence at different time points, as well as any mention to the magnitude of the fall in incidence (e.g., rates have halved).

Table S3b. Information items about cervical screening benefits, risks, results, and cervical cancer statistics (Part 2)

	Marie Stopes UK	NHS Choices (England)	NHS Inform (Scotland)	Patient Info	PHA Northern Ireland	Public Health Wales	Women's Health Concern
**Screening benefits**							
Risk reduction of developing cervical cancer	RR	N	RR	RR	RR	RR	-
Risk reduction of death from cervical cancer	-	-	-	-	-	-	-
Risk reduction of total mortality	-	-	-	-	-	-	-
* Lives saved yearly	N	N	N	N	-	N	N
* Fall in incidence since the introduction of screening [a]	-	N	-	N	-	N	-
**Screening risks**							
Overdiagnosis/overtreatment	0	V	V	V, N	-	V, 1X	V
Pain/discomfort related to the test	V	V	-	V	V	V	0
False negative results	-	V	V	V	V	V	V, N
Psychological distress due to abnormal results	-	0	-	0	-	-	-
False positive results	-	V	0	0	-	V	-
Risks related to treatment	-	V	-	-	0	V	-
**Screening results**							
Possibility of inadequate result	0	V, N	V	N	V, N	1X, N	V
Possibility of abnormal result	0	V, 1X, N, G	V, 1X, G	V, N	V, 1X, N	V, 1X, N	-
Positive predictive value	-	-	-	-	-	-	-
* Possibility of cancer diagnosis	-	V, 1X	-	V, 1X	-	V	-
**Cervical cancer statistics**							
Survival from cervical cancer	-	-	-	-	-	-	-
Lifetime risk of developing cervical cancer	-	-	-	-	-	-	-
Lifetime risk of dying from cervical cancer	-	-	-	-	-	-	-
* Cervical cancer incidence	N	N	-	N	N	N	-
* Cervical cancer mortality	-	-	-	-	N	N	-
**Type of appeals**							
Participation	-	-	x	x	x	x	x
Informed decision making	-	x	x	-	-	x	x

Note: - = item not present; 0 = not quantified; V = verbal quantifier; RR = numerical relative risk reduction; 1X = numerical 1-in-X; N = numerical (other); G = graph; x = item present (coding format not applicable); * codes added by the authors; [a] includes estimates of incidence at different time points, as well as any mention to the magnitude of the fall in incidence (e.g., rates have halved).
