# Supplementary material for: How is cervical cancer screening information communicated in UK websites? Cross-sectional analysis of content and quantitative presentation formats
Source: BMJ Open. 2019 Oct 28;9(10):e029551. doi: 10.1136/bmjopen-2019-029551 (PMC6830680; doi:10.1136/bmjopen-2019-029551)
Supplement: Author's manuscript [file bmjopen-2019-029551.draft_revisions.pdf]

BMJ Open

BMJ Open is committed to open peer review. As part of this commitment we make the peer review history of every article we publish publicly available.

When an article is published we post the peer reviewers' comments and the authors' responses online. We also post the versions of the paper that were used during peer review. These are the versions that the peer review comments apply to.

The versions of the paper that follow are the versions that were submitted during the peer review process. They are not the versions of record or the final published versions. They should not be cited or distributed as the published version of this manuscript.

BMJ Open is an open access journal and the full, final, typeset and author-corrected version of record of the manuscript is available on our site with no access controls, subscription charges or pay-per-view fees (http://bmjopen.bmj.com).

If you have any questions on BMJ Open's open peer review process please email info.bmjopen@bmj.com

BMJ Open

How is cervical cancer screening information communicated in UK websites?

Journal:	*BMJ Open*
Manuscript ID	bmjopen-2019-029551
Article Type:	Research
Date Submitted by the Author:	30-Jan-2019
Complete List of Authors:	Okan, Yasmina; University of Leeds, Centre for Decision Research, Leeds University Business School<br>Smith, Sam; University of Leeds, Leeds Institute of Health Sciences<br>Bruine de Bruin, Wändi; University of Leeds, Centre for Decision Research, Leeds University Business School; Carnegie Mellon University, Department of Engineering and Public Policy
Keywords:	cervical cancer screening, informed decision making, risk communication, risk perception, web analysis

SCHOLARONE™
Manuscripts

Running Head: CERVICAL CANCER SCREENING WEBSITES

Word count: 3,494

How is cervical cancer screening information communicated in UK websites?

Yasmina Okan,[1] * Samuel G. Smith,[2] Wändi Bruine de Bruin,[1,3]

[1]Centre for Decision Research, Leeds University Business School, University of Leeds, Leeds, UK

[2] Leeds Institute of Health Sciences, University of Leeds, Leeds, UK

[3]Department of Engineering and Public Policy, Carnegie Mellon University, Pittsburgh, US

* Corresponding author at Centre for Decision Research, Leeds University Business School, University of Leeds, Charles Thackrah Building, Leeds, LS2 9LB, UK; E-mail: y.okan@leeds.ac.uk, Phone: +44 113 343 2622.

For peer review only

Abstract

Objectives: To investigate whether UK websites about cervical cancer screening targeted to the public include (1) information about benefits and risks of screening, possible screening outcomes, and cervical cancer statistics, (2) formats recommended in the risk communication literature, and (3) appeals for participation and/or informed decision making.

Design: Cross-sectional analysis of websites using a checklist of information items.

Outcome measures: We recorded the number of websites that contained each of the information items, and the presentation format used for probabilistic information (no quantification provided, verbal quantifiers only, different types of numerical formats, and/or graphs). We also recorded the number of websites that contained appeals for participation and/or references to informed decision making.

Setting: Websites identified using the most common Google search terms used in the UK to find information on cervical screening, based on GoogleTrends and a commercial internet-monitoring programme. Two additional websites were identified through the authors' knowledge of resources on cervical screening.

Results: After applying exclusion criteria, 14 websites were evaluated, including websites of public and private health service providers, charities, a medical society, and a pharmacy. Content of the websites included different benefits, risks of screening, and possible outcomes. However, specific content varied across websites. Probabilistic information was often presented using non-recommended formats, including relative risk reductions to express screening benefits, and verbal quantifiers without numbers to express risks. Appeals for participation without mention to informed decision making were present in half of the websites.

Conclusions: UK websites about cervical cancer screening may not fully support informed decision making about screening participation. Although websites were generally balanced,

For peer review only

the specific content varied, and recommendations from the literature to facilitate understanding of quantitative information were often not followed. Designing websites that adhere to existing recommendations may support informed cervical screening uptake.

Keywords: cervical cancer screening; informed decision making; risk communication; risk perception; web analysis

For peer review only

Article summary

Strengths and limitations of this study

- We performed a comprehensive analysis of the content of UK websites about cervical cancer screening using an established checklist of information items, and identified additional information that was mentioned commonly.
- This was the first study to systematically examine whether cancer screening websites present probabilistic information using formats recommended in the risk communication literature.
- We identified websites using the most common Google search terms used in the UK to find information on cervical screening, and examined the majority of links that users will realistically access.
- All the information items we assessed may not be relevant for screening decisions, whereas we may have omitted some information that can be relevant (e.g., information about the human papillomavirus).
- We did not examine whether websites mentioned the uncertainty existing around estimates of benefits and risks, although such information can be important for informed decision making.

For peer review only

INTRODUCTION

Cervical cancer is highly preventable. It is caused by an infection with the human papillomavirus (HPV), which may lead to abnormal changes in the cells of the cervix. Such abnormalities can be detected through screening and treated before they become cancerous. There is substantial evidence that cervical screening significantly reduces both cervical cancer incidence and cancer-specific mortality[1–5]. Indeed, in countries with established screening programs, cervical cancer has become relatively rare. In the UK, age-standardized incidence was 11.8 in 100,000 in 2014, and age-standardized mortality was 3.3 in 100,000[6]. In England alone, estimates suggest that there would be 1,827 additional cervical cancer deaths per year without screening[5].

Despite its well-documented benefits, cervical screening is also associated with potential risks. These include the detection and treatment of abnormal cells that would have cleared up on their own (i.e., overdiagnosis and overtreatment[7,8]), and an increased risk of preterm birth among women who received treatment, which involves the removal of abnormal cells[9–11]. A recent meta-analysis indicates that about half of untreated moderately abnormal cells regress within two years[7], implying that overtreatment may occur among screening participants. However, experts and policy makers tend to agree that benefits of cervical screening outweigh potential risks[2,12,13]. In the UK, the National Health Service offers screening to all women aged 25 to 64. It has been emphasized that screening invitees need information on both benefits and risks to make informed decisions about participation[12,14,15].

Organized screening programs often rely on written materials to inform the public, including web-based communications. In the UK, eligible women receive an invitation accompanied by an information leaflet and links to different websites about cervical screening. Hence in this context it becomes particularly relevant to examine whether widely

For peer review only

accessed websites achieve the intended goal of supporting decisions about screening participation. Both European and UK guidelines have emphasized that cancer screening communications should be comprehensive in content, and should provide a balanced discussion of benefits and risks to screening, as well as potential outcomes[15–18]. Such guidelines have also highlighted that screening communications should be comprehensible, and avoid presentations of quantitative information that are hard to understand (e.g., about screening benefits and risks[15–17]). Communications that are not well understood can lead to undue concern and undermine informed uptake[19,20]. This can be particularly problematic for individuals with lower health literacy or numeracy, ultimately contributing to inequalities in screening participation[21,22].

Quantitative information in screening communications, however, can be challenging even for educated audiences[19,23]. Such information can be presented using a range of different formats, including verbal quantifiers (e.g., “low” or “moderate” risk), different types of numerical formats, and graphical displays (e.g., icon arrays). Research in risk communication has shown that presentation formats vary considerably in their effectiveness to promote accurate understanding. Although evidence concerning the effectiveness of some formats is still limited[24], other formats have shown strong evidence for either improving or hindering understanding. Table 1 provides an overview of such formats and associated evidence-based recommendations. Key recommendations relevant to screening communications include to avoid the use of verbal quantifiers without accompanying numbers; numerical “1-in-X” formats; and presentations of risk reductions in relative terms. Another common recommendation is to add simple graphical displays to numerical information.

For peer review only

Table 1. Evidence-based recommendations from the risk communication literature to promote understanding of risk and benefit information

Recommendation	Rationale	Key references (first author)
Avoid the use of verbal quantifiers without numbers (e.g., women who have abnormal cells removed are *slightly more likely* to have their baby early”).	Interpretations of verbal quantifiers vary across individuals and often differ from interpretations intended by communicators. Verbal quantifiers can lead to overestimations of risks.	Budescu [60]; Knapp [46,47]; Peters [45]; Visschers et al. [48]; Young [44]; Zipkin [33]
Avoid numerical “1-in-X” formats to present the chance of an outcome (e.g., 1 in 12 women will have an abnormal test result).	People tend to perceive the same probabilities as higher and more worrying when presented in a “1-in-X” format, as compared to numerically equivalent “N-in-X*N” ratios (e.g., 10 in 120 women will have an abnormal test result) or percentages (e.g., 5% women will have an abnormal test result).	Pighin [61,62]; Sirota [43,63,64]; Trevena [42]; Zikmund-Fisher [34]
Avoid presenting estimates of risk reduction in relative terms (e.g. screening cuts the risk of getting cervical cancer by 75%).	Relative risk differences can obscure the true magnitude of benefit or harm, as compared to absolute risk differences (e.g., screening reduces the risk of getting cervical cancer from 20 in 1,000 to 5 in 1,000).	Akl [65]; Covey [66]; Fagerlin [20], Gigerenzer [19,67]; Trevena [42] Zipkin [33]
Add simple graphical displays to numerical information (e.g., icon arrays, where icons of different colors represent those affected and not affected by the risk).	Well-designed, simple graphs help to overcome difficulties understanding numerical information and are often perceived as more appealing and easier to understand.	Galesic [51]; Garcia-Retamero [35]; Gigerenzer [67]; Okan [68]; Paling [69]; Schapira [70]

For peer review only

Finally, existing guidelines have also stated that screening communications should not aim to persuade people to attend or present screening as necessary or important, without acknowledging that not participating is a reasonable choice[15–18]. For instance, recent guidance from the UK National Screening Committee[15] notes specifically that "information should make it clear that it is a personal choice to accept or decline screening and both choices will be fully supported" (p. 6). At the heart of this notion lies the idea of respecting personal autonomy and ensuring that screening decisions are in line with invitees' personal values and circumstances.

We aimed to investigate whether UK websites presenting information about cervical screening adhere with existing guidelines and policy recommendations concerning informational content and type of appeals (i.e., appeals for participation vs. informed decision making), as well as with recommendations from the risk communication literature concerning presentation format. Previous website analyses have primarily focused on breast cancer screening, and have either assessed only website content[25,26], or specific aspects of presentation format (e.g., consistency in the presentation of statistics on overdiagnosis[27]). We used an established checklist of cervical screening benefits, risks, and outcomes, as well as cervical cancer statistics[28], building on earlier work examining breast cancer screening communications[25,29,30]. We also examined whether the websites contained additional information that was mentioned commonly, and that may be relevant for screening decisions. We assessed presentation format considering the recommendations listed in Table 1. Finally, we examined whether websites contained appeals for participation and/or informed decision making. In sum, our research questions were:

1. Do UK websites about cervical screening contain a) key information about screening benefits and risks, possible outcomes, and b) cervical cancer statistics?

For peer review only - http://bmjopen.bmj.com/site/about/guidelines.xhtml

2. Do UK websites about cervical screening present probabilistic information using recommended formats?

3. Do UK websites about cervical screening contain appeals for participation and/or informed decision making?

METHODS

Search strategy

We used GoogleTrends to identify the most common Google search terms used in the UK to find information on cervical screening. We identified search terms related to “cervical screening” in the 12 months prior to March 2017. We used “cervical screening” as it is used in the UK’s National Health Service invitation to get screened. Related search terms included “cervical cancer screening”, “cervical smear” and “smear test.” To identify which of these terms had been used more commonly, we next used Wordtracker (https://app.wordtracker.com/), a commercial program that estimates the relative frequency of Google search terms in a given period of time (see[31] for a similar procedure). The two most commonly used term in the UK during the 12 months prior to March 2017 had been “smear test”, followed by “cervical screening”.

Website selection

On March 9th, 2017, we performed the Google search ‘“smear test” OR “cervical screening”’ using the private Firefox browsing mode to prevent previously visited pages and cookies from influencing search results. Following Ghanouni et al.,[27] we examined the first five pages of results using the default number of results per page (i.e., 10 per page), as this includes the vast majority of websites that users will access (see also[32]). This resulted in 50 links examined initially. We excluded links that (1) were aimed at healthcare professionals and academics rather than lay people (e.g., technical reports, research articles), (2) reflected press releases or media articles (e.g., online newspapers, magazines), (3) were locally or

For peer review only - http://bmjopen.bmj.com/site/about/guidelines.xhtml

regionally-oriented (e.g., discussing logistics for specific GP surgeries or Clinical Commissioning Groups), (4) presented information not applicable to the UK due to an international focus, (5) were duplicate, (6) included no or little information about cervical screening (fewer than 150 words), (7) included no written materials (e.g., YouTube video). Figure 1 shows results of the process of website selection, which are discussed in detail below.

For each of the selected websites, we coded all the information that appeared in the link identified through the Google search. We also coded sections on cervical screening within each website that could be directly accessed from the main link identified through the search, as well as other written materials that were directly accessible, including electronic leaflets, fact sheets, and slideshows. In the analysis reported below, written materials that could be accessed through a given website were considered as part of the same resource. Websites were accessed between 20th March 2017 and 28th April 2017, with the exception of one website (BootsWebMD), which was accessed at a later date due to an oversight following the initial website selection. The specific date of access for each website and corresponding links can be found in online Supplementary materials (Table S1). Copies of PDF files reflecting the content of all websites at the time of access are available at the Open Science Framework (https://osf.io/73gfn/?view_only=1dbf5d9e16c64b2aaa2cd3af704e7103).

Coding of websites

To code website content, we adapted a checklist of information items that was developed to analyze invitations for cervical screening[28]. Five additional information codes were generated following an initial inspection of the websites, representing information that was mentioned commonly, and may be relevant for screening decisions (e.g., additional benefits not reflected in the original checklist). Examples corresponding to all the information items can be found in online Supplementary materials (Table S2).

For peer review only

To assess presentation format, we coded whether items involving probabilistic information were presented using: no quantifiers, verbal quantifiers only, numerical quantifiers (1-in-X vs. other numerical format), and/or graphs. For items concerning risk reduction we also coded whether estimates were presented in relative terms. Multiple formats (e.g., graphs accompanied by numbers) were present in some communication materials (see online Supplementary materials Table S3).

Finally, to assess type of appeal we coded references to informed decision making (e.g., "deciding whether to have screening or not is your choice") and appeals for participation. Following Kotlhoff et al.[28], the latter category included direct encouragement for participation (e.g., "a smear test—have it") and statements presenting screening as necessary (e.g., "it's essential for women to have regular cervical screening tests") or important (e.g., "it is important to go for screening every time you are invited").

One of the authors (YO) read through all the selected materials and applied the coding scheme above. Three websites (> 20%) were coded by another author (SS). Cohen's kappa was moderate to high on all codes except for article tone (.18). An inspection revealed that discrepancies for this code were caused by oversight of appeals for participation that were not very prominent. These discrepancies were resolved after discussion among the two coders, resulting in complete agreement for this code. The resulting average Cohen's kappa was .86 (range: .65 to 1).

RESULTS

Website selection

The first five pages of results of the Google search included 50 websites. After applying exclusion criteria 12 websites remained (see Figure 1), including four websites from public health service providers (NHS Choices England, NHS Inform Scotland, Public Health Agency Northern Ireland, Public Health Wales) one private health service provider (Bupa

UK), three cancer charities (Cancer Research UK, Jo's Cervical Cancer Trust, MacMillan Cancer Support), two additional charities (LGBT Foundation, Marie Stopes UK), one medical society (British Society for Colposcopy and Cervical Pathology), and one pharmacy (Boots WebMD). Two additional websites (Patient and Women's Health Concern) were identified through the authors' knowledge of resources on cervical screening. Below we present summary statistics across websites. Results for individual websites can be found in online Supplementary materials (Table S3).

<Figure 1>

Do UK websites about cervical screening contain a) key information about screening benefits and risks, possible outcomes, and b) cervical cancer statistics?

We report on four main findings regarding website content. First, screening benefits were mentioned frequently, although the focus was primarily on risk reduction for developing cervical cancer (Figure 2a). Estimates of risk reduction varied across and sometimes within websites and ranged between 60% and 90%. Risk reductions of cervical or total mortality were not mentioned explicitly on any website. However, benefits were often expressed in terms of the number of lives saved yearly (e.g. "cervical screening saves 5,000 lives a year in the UK"; n=10 websites) or the fall in cervical cancer cases since the national screening programme was introduced (e.g., "since the screening programme was introduced in the 1980s, the number of cervical cancer cases has decreased by about 7% each year"; n=6).

Second, screening risks were also mentioned relatively frequently (Figure 2b), but the specific risks mentioned and how these were described varied across websites. The most commonly mentioned risks were overdiagnosis and overtreatment (n=12), pain/discomfort related to the cytology test (n=12), and the possibility of false negatives (n=10). Descriptions of overdiagnosis and overtreatment generally focused on the fact that cell changes often clear up and won't progress into cancer (e.g., "in most cases, the abnormal cells will disappear on

For peer review only

their own”). Based on this description alone, women may not be able to infer that cervical screening may lead to unnecessary treatment. The risk for unnecessary additional tests or treatment was only mentioned explicitly in seven cases, often in connection to justifications for current screening age ranges. Most websites noted that the test may be uncomfortable but is generally not painful (e.g. “having the speculum put in may be a little uncomfortable, but it shouldn’t hurt”), although one website did describe the test as potentially painful. Risks related to treatment were only mentioned in half of the websites (Figure 2b), all of which mentioned the possibility of premature birth. Two websites also referred to the risk of stenosis (i.e., the cervix becoming tightly closed after treatment).

Third, possible screening outcomes were mentioned in most websites (Figure 2c). Estimates of the likelihood of inadequate results varied between 1.7 and 3% and estimates of the likelihood of abnormal results ranged between 5 and 10%. Additionally, the possibility of cancer diagnosis was mentioned in over half of the websites (n=8) and was often reported to occur in less than 1 in 1,000 cases.

Finally, cervical cancer statistics such as lifetime risk of developing or dying from cervical cancer were seldom discussed, although some websites did provide details on cervical cancer incidence and mortality (Figure 2d). Specific estimates of incidence again varied across websites, and included yearly, daily, national, and regional estimates (e.g., “each year in Northern Ireland, about 103 women are diagnosed with cervical cancer”).

<Figures 2a, 2b, 2c, and 2d>

Do UK websites about cervical screening present probabilistic information using recommended formats?

Information was often not presented in recommended formats. First, the recommendation to avoid the use of verbal quantifiers without numbers[33] was often not met for information about risks (Figure 2b). Specifically, risks related to treatment were quantified only verbally

For peer review only

in all websites, though the specific verbal quantifiers varied. For example, one website stated that "women are *slightly more likely* to have their baby 1 to 2 months early", whereas another one stated that "women may have a *higher risk* of preterm delivery". Other risks were also either only quantified verbally or not quantified at all, with the exception of overdiagnosis/overtreatment (n=3) and false negative results (n=2).

Second, the recommendation to avoid "1-in-X" numerical formats[34] was in most cases violated for information about screening outcomes. In fact, this was the most popular format to convey the likelihood of abnormal results (Figure 2c). Third, the recommendation to avoid presentations of relative risk reduction[19] was violated in most cases in which an estimate of risk reduction was provided (Figure 2a). Finally, the recommendation to add simple graphical displays[35] was also often not met. Only two websites contained graphs, which in both cases depicted the likelihood of abnormal results.

Do UK websites about cervical screening contain appeals for participation and/or informed decision making?

Appeals for participation were present in most websites (n=12). Specifically, appeals for participation without mention to informed decision making were present in half of the websites (n=7). This contrasts with existing guidelines stating that communications should acknowledge that it is reasonable to decline the offer[12,15,18]. A combination of appeals for participation and references to informed decision making was present in several websites (n=5) (e.g., "don't ignore your smear test, it could save your life· and "taking part in cervical screening is your choice"). Finally, one website referred to informed decision making only ("deciding whether or not to have a cervical screening test is your choice").

DISCUSSION

We investigated whether UK websites about cervical screening adhere with existing recommendations concerning content, presentation formats, and type of appeals. We found

For peer review only - http://bmjopen.bmj.com/site/about/guidelines.xhtml

that websites often followed recommendations in European and UK guidelines to include information about both benefits and risks of screening[15–17]. However, specific content varied across websites. For instance, only half of the websites explicitly referred to the risks related to the treatment of abnormal cells. Additionally, probabilistic information was often presented using formats not recommended in the risk communication literature, including relative risk reductions to express screening benefits, and verbal quantifiers without numbers to express risks. The non-recommended numerical format "1-in-X" was also used often to express possible screening outcomes (e.g., 1 in 12 women will have an abnormal test result). Moreover, we found that several websites encouraged screening participation without referring to informed decision making. This contrasts with current UK policy, which emphasizes that communications can recommend people to participate in screening, but should acknowledge that not accepting the offer is a reasonable choice[12,18,36].

Interestingly, our finding that websites often included information on both benefits and risks of cervical screening contrasts with previous analyses of breast cancer screening websites. Specifically, previous work documented a lack of information about breast screening risks such as overdiagnosis[25,26]. It is likely that recent emphasis in the UK on facilitating informed screening decisions[12,36] has led to more comprehensive and balanced websites in recent years (see also[27]). The presence of information on both screening benefits and risks is aligned with women's preferences, who often want to receive information on both aspects before their test[37]. However, our finding that the specific content varied across websites suggests that women accessing different resources may come to different conclusions about screening. Moreover, existing descriptions of benefits and risks may not always support understanding. For instance, the concepts of overdiagnosis and overtreatment are often unfamiliar and counterintuitive for people[38–40], highlighting the need to provide explanations that are accessible and transparent. We also found that many

For peer review only

websites emphasized that screening “saves lives”. It has been argued that such claim can be misleading, and that communications should report reductions in cancer specific mortality, overall mortality, as well as overall cancer deaths[41].

The finding that probabilistic information was often presented using non-recommended formats is concerning as this may lead to misperceptions, even among educated audiences. The use of relative risk reductions to express screening benefits can hinder understanding and increase risk perceptions, relative to presentations of absolute risk reduction[33,42]. At the same time, the use of “1-in-X” formats to express the likelihood of abnormal cervical screening results may lead to overestimations of the likelihood of such results and undue worry[43]. Similarly, the use of verbal quantifiers without numbers to express cervical screening risks may lead women to overestimate such risks[44–47], potentially deterring them from screening participation. The absence of numerical information can also lead people to perceive communications as less credible and trustworthy[48–50]. Although some risks are hard to quantify (e.g., psychological distress), estimates are available for risks such as preterm birth or overtreatment, based on observational studies and meta-analyses[7,9,11]. Simple, well-designed graphs could potentially be used to communicate such estimates in accessible ways[35,51]. This could be particularly beneficial for people with low numeracy, who tend to struggle to use numerical information when forming perceptions of risks and benefits[52,53]. Yet, our analysis also revealed that graphs are seldom used in cervical screening websites.

Strengths of our work include that our analysis was comprehensive and systematically examined website content using an established checklist of information items[28], as well as presentation formats, considering recommendations from the literature. As noted earlier, previous website analyses either assessed only website content[25,26], or specific aspects of presentation format[27]. Our work provides a framework that could be used by researchers

For peer review only - http://bmjopen.bmj.com/site/about/guidelines.xhtml

and practitioners to evaluate the content and format of websites about related topics (e.g., other types of screening). Additionally, our analysis included most of the websites that UK citizens may realistically access, as our strategy for website selection was based on the most commonly used Google search terms, and we examined the first five pages of results. Limitations of our study include that our analyses may not have covered all information that is potentially relevant for screening decisions. For instance, we did not assess whether information about the human papillomavirus (HPV) was included in websites. Conversely, it is also possible that all codes we assessed are not essential for making informed screening decisions. Finally, we did not examine whether websites mentioned the uncertainty existing around estimates of benefits and risks. Communicating uncertainty to laypeople can be challenging[54–56], but is important to promote informed decision making[14,15,36].

Future work should aim to identify essential information items for informed decisions about screening, considering both experts' and women's views. As noted by Ghanouni et al.[57], guidelines often do not provide detailed advice on *which* specific screening benefits, risks, or screening outcomes should be discussed in communications. Moreover, experts may not fully agree on the relative importance of different information items, and women may have different information needs and preferences, including about whether and when to receive information about further tests. Providing too much unfamiliar information initially may also overwhelm screening invitees, and potentially distract them from key information necessary for decision making[20,58]. Future work should also examine the relative effectiveness of different presentation formats, and potential interactions with different information items. Randomized trials have started to examine how different presentation formats can affect cervical screening decisions[59], but such evidence is still relatively scarce, and additional work is needed. Such work would provide valuable insights to inform the design of evidence-based public communications about screening.

For peer review only

Acknowledgements

We are grateful to Mirta Galesic and Erika Waters for their valuable comments and suggestions.

Funding

This work was supported by a Population Research Fellowship awarded by Cancer Research UK to Yasmina Okan (reference C57775/A22182). Samuel G. Smith is supported by Yorkshire Cancer Research. Wändi Bruine de Bruin was in part supported by a grant from the Swedish Riksbanken Jubilieumsfond programme on Science and Proven Experience. The funding agencies had no involvement in designing the study, data collection, analysis and interpretation, writing the report, or the decision to submit the article for publication.

Competing interests

SS is an academic consultant for Luto, who are not involved in any of the websites reviewed in this manuscript.

Author contributions

YO conceived the study and all authors contributed to design it. YO acquired and analysed the data, and SS assisted with data analyses. All authors contributed to data interpretation. YO drafted the initial version of the manuscript, and all authors revised it critically and approved the final version.

Data sharing statement

Copies of PDF files reflecting the content of all websites at the time of access are available at the Open Science Framework (https://osf.io/73gfn/?view_only=1dbf5d9e16c64b2aaa2cd3af704e7103).

For peer review only

References

1 Nygård M. Screening for cervical cancer: when theory meets reality. *BMC Cancer* 2011;11:240.

2 Esserman LJ, Thompson IM, Reid B. Overdiagnosis and overtreatment in cancer: an opportunity for improvement. *J Am Med Assoc* 2013;310:797–798.

3 Peto J, Gilham C, Fletcher O, *et al.* The cervical cancer epidemic that screening has prevented in the UK. *Lancet* 2004;364:249–256.

4 Peirson L, Fitzpatrick-Lewis D, Ciliska D, *et al.* Screening for cervical cancer: A systematic review and meta-analysis. *Syst Rev* 2013;2:35.

5 Landy R, Pesola F, Castañón A, *et al.* Impact of cervical screening on cervical cancer mortality: Estimation using stage-specific results from a nested case–control study. *Br J Cancer* 2016;115:1140–1146.

6 Smittenaar CR, Petersen KA, Stewart K, *et al.* Cancer incidence and mortality projections in the UK until 2035. *Br J Cancer* 2016;115:1147–1155.

7 Tainio K, Athanasiou A, Tikkinen KA, *et al.* Clinical course of untreated cervical intraepithelial neoplasia grade 2 under active surveillance: Systematic review and meta-analysis. *Br Med J* 2018;360:k499.

8 McCredie MR, Sharples KJ, Paul C, *et al.* Natural history of cervical neoplasia and risk of invasive cancer in women with cervical intraepithelial neoplasia 3: A retrospective cohort study. *Lancet Oncol* 2008;9:425–434.

9 Castanon A, Landy R, Brocklehurst P, *et al.* Risk of preterm delivery with increasing depth of excision for cervical intraepithelial neoplasia in England: Nested case-control study. *Br Med J* 2014;349:g6223.

For peer review only

10 Sasieni P, Castanon A, Landy R, *et al.* Risk of preterm birth following surgical treatment for cervical disease: executive summary of a recent symposium. *BJOG Int J Obstet Gynaecol* 2016;123:1426–1429.

11 Kyrgiou M, Athanasiou A, Kalliala IEJ, *et al.* Obstetric outcomes after conservative treatment for cervical intraepithelial lesions and early invasive disease. *Cochrane Database Syst Rev* 2017;11:CD012847.

12 Forbes LJ, Ramirez AJ. Communicating the benefits and harms of cancer screening. *Curr Oncol Rep* 2014;16:382.

13 Moyer VA. Screening for cervical cancer: US Preventive Services Task Force recommendation statement. *Ann Intern Med* 2012;156:880–891.

14 Hersch JK, Nickel BL, Ghanouni A, *et al.* Improving communication about cancer screening: Moving towards informed decision making. *Public Health Res Pract* 2017;27:e2731728.

15 UK National Screening Committee. Guidance for the development, production and review of information to support UK population screening programmes. 2018. https://www.gov.uk/government/publications/uk-national-screening-committee-information-development-guidance (accessed 17 Aug 2018).

16 European Commission. *European guidelines for quality assurance in cervical cancer screening. 2nd edition.* Luxembourg: : Office for Official Publications of the European Communities 2008. http://screening.iarc.fr/doc/ND7007117ENC_002.pdf (accessed 17 Aug 2018).

17 General Medical Council. Consent: patients and doctors making decisions together. 2008. https://www.gmc-uk.org/ethical-guidance/ethical-guidance-for-doctors/consent (accessed 17 Aug 2018).

For peer review only

18 Entwistle VA, Carter S, Trevena L, *et al.* Communicating about screening. *Br Med J* 2008;333:789–91.

19 Gigerenzer G, Gaissmaier W, Kurz-Milcke E, *et al.* Helping doctors and patients make sense of health statistics. *Psychol Sci Public Interest* 2007;8:53–96.

20 Fagerlin A, Zikmund-Fisher BJ, Ubel PA. Helping patients decide: ten steps to better risk communication. *J Natl Cancer Inst* 2011;103:1436–1443.

21 Smith SG, Kobayashi LC, Wolf MS, *et al.* The associations between objective numeracy and colorectal cancer screening knowledge, attitudes and defensive processing in a deprived community sample. *J Health Psychol* 2016;21:1665–1675.

22 Smith SG, McGregor LM, Raine R, *et al.* Inequalities in cancer screening participation: examining differences in perceived benefits and barriers. *Psychooncology* 2016;25:1168–1174.

23 Schwartz LM, Woloshin S, Black WC, *et al.* The role of numeracy in understanding the benefit of screening mammography. *Ann Intern Med* 1997;127:966–972.

24 Spiegelhalter D. Risk and uncertainty communication. *Annu Rev Stat Its Appl* 2017;4:31–60.

25 Jørgensen KJ, Gøtzsche PC. Presentation on websites of possible benefits and harms from screening for breast cancer: Cross sectional study. *Br Med J* 2004;328:148.

26 Attena F, Cancellieri M, Pelullo CP. Scarce information about breast cancer screening: An Italian websites analysis. *Medicine (Baltimore)* 2016;95:e5615.

27 Ghanouni A, Meisel SF, Hersch J, *et al.* Information on “overdiagnosis” in breast cancer screening on prominent United Kingdom-and Australia-oriented health websites. *PLoS One* 2016;11:e0152279.

For peer review only

28 Kolthoff SK, Hestbech MS, Jørgensen KJ, *et al.* Do invitations for cervical screening provide sufficient information to enable informed choice? A cross-sectional study of invitations for publicly funded cervical screening. *J R Soc Med* 2016;109:274–281.

29 Slaytor EK, Ward JE. How risks of breast cancer and benefits of screening are communicated to women: Analysis of 58 pamphlets. *Br Med J* 1998;317:263–264.

30 Jørgensen KJ, Gøtzsche PC. Content of invitations for publicly funded screening mammography. *Br Med J* 2006;332:538–541.

31 McHugh SM, Corrigan M, Morney N, *et al.* A quantitative assessment of changing trends in internet usage for cancer information. *World J Surg* 2011;35:253–257.

32 Liebl P, Seilacher E, Koester M-J, *et al.* What cancer patients find in the internet: The visibility of evidence-based patient information - Analysis of information on German websites. *Oncol Res Treat* 2015;38:212–218. doi:10.1159/000381739

33 Zipkin DA, Umscheid CA, Keating NL, *et al.* Evidence-based risk communication: a systematic review. *Ann Intern Med* 2014;161:270–280.

34 Zikmund-Fisher BJ. Continued use of 1-in-X risk communications is a systemic problem. *Med Decis Making* 2014;34:412–413.

35 Garcia-Retamero R, Cokely ET. Designing visual aids that promote risk literacy: A systematic review of health research and evidence-based design heuristics. *Hum Factors* 2017;59:582–627.

36 Forbes LJ, Ramirez A-J, list) E group on I about BS. Offering informed choice about breast screening. *J Med Screen* 2014;21:194–200.

37 Dieng M, Trevena L, Turner RM, *et al.* What Australian women want and when they want it: Cervical screening testing preferences, decision-making styles and information needs. *Health Expect* 2013;16:177–188.

For peer review only

38 Waller J, Whitaker KL, Winstanley K, *et al.* A survey study of women's responses to information about overdiagnosis in breast cancer screening in Britain. *Br J Cancer* 2014;111:1831–1835.

39 Nagler RH, Franklin Fowler E, Gollust SE. Women's awareness of and responses to messages about breast cancer overdiagnosis and overtreatment. *Med Care* 2017;55:879–885.

40 Ghanouni A, Renzi C, Waller J. Improving public understanding of 'overdiagnosis' in England: A population survey assessing familiarity with possible terms for labelling the concept and perceptions of appropriate terminology. *BMJ Open* 2018;8:e021260.

41 Gigerenzer G. Full disclosure about cancer screening. *Br Med J* 2016;352:h6967.

42 Trevena LJ, Zikmund-Fisher BJ, Edwards A, *et al.* Presenting quantitative information about decision outcomes: a risk communication primer for patient decision aid developers. *BMC Med Inform Decis Mak* 2013;13:S7.

43 Sirota M, Juanchich M, Bonnefon J-F. 1-in-X" bias: "1-in-X" format causes overestimation of health-related risks. *J Exp Psychol Appl* Published Online First: 2018. doi:10.1037/xap0000190

44 Young S, Oppenheimer DM. Effect of communication strategy on personal risk perception and treatment adherence intentions. *Psychol Health Med* 2009;14:430–442.

45 Peters E, Hart PS, Tusler M, *et al.* Numbers matter to informed patient choices: A randomized design across age and numeracy levels. *Med Decis Making* 2014;34:430–442.

46 Knapp P, Raynor DK, Berry DC. Comparison of two methods of presenting risk information to patients about the side effects of medicines. *BMJ Qual Saf* 2004;13:176–180.

For peer review only

47 Knapp P, Gardner PH, Woolf E. Combined verbal and numerical expressions increase perceived risk of medicine side-effects: A randomized controlled trial of EMA recommendations. *Health Expect* 2016;19:264–274.

48 Visschers VH, Meertens RM, Passchier WW, *et al.* Probability information in risk communication: A review of the research literature. *Risk Anal* 2009;29:267–287.

49 Jenkins SC, Harris AJ, Lark RM. When unlikely outcomes occur: the role of communication format in maintaining communicator credibility. *J Risk Res* 2018.

50 Gurmankin AD, Baron J, Armstrong K. The effect of numerical statements of risk on trust and comfort with hypothetical physician risk communication. *Med Decis Making* 2004;24:265–271.

51 Galesic M, Garcia-Retamero R, Gigerenzer G. Using icon arrays to communicate medical risks: Overcoming low numeracy. *Health Psychol* 2009;28:210–216.

52 Dieckmann NF, Slovic P, Peters EM. The use of narrative evidence and explicit likelihood by decisionmakers varying in numeracy. *Risk Anal* 2009;29:1473–1488.

53 Lipkus IM, Peters E, Kimmick G, *et al.* Breast cancer patients' treatment expectations after exposure to the decision aid program adjuvant online: The influence of numeracy. *Med Decis Making* 2010;30:464–473.

54 Longman T, Turner RM, King M, *et al.* The effects of communicating uncertainty in quantitative health risk estimates. *Patient Educ Couns* 2012;89:252–259.

55 Sladakovic J, Jansen J, Hersch J, *et al.* The differential effects of presenting uncertainty around benefits and harms on treatment decision making. *Patient Educ Couns* 2016;99:974–980.

56 Han PK. Conceptual, methodological, and ethical problems in communicating uncertainty in clinical evidence. *Med Care Res Rev* 2013;70:14S–36S.

For peer review only

57 Ghanouni A, Renzi C, Meisel SF, *et al.* Common methods of measuring 'informed choice'in screening participation: Challenges and future directions. *Prev Med Rep* 2016;4:601–607.

58 Peters E, Dieckmann N, Dixon A, *et al.* Less is more in presenting quality information to consumers. *Med Care Res Rev* 2007;64:169–190.

59 Hestbech MS, Gyrd-Hansen D, Kragstrup J, *et al.* Effects of numerical information on intention to participate in cervical screening among women offered HPV vaccination: A randomised study. *Scand J Prim Health Care* 2016;34:401–419.

60 Budescu DV, Por H-H, Broomell SB, *et al.* The interpretation of IPCC probabilistic statements around the world. *Nat Clim Change* 2014;4:508–512.

61 Pighin S, Savadori L, Barilli E, *et al.* The 1-in-X effect on the subjective assessment of medical probabilities. *Med Decis Making* 2011;31:721–729.

62 Pighin S, Savadori L, Barilli E, *et al.* Communicating Down syndrome risk according to maternal age: "1-in-X" effect on perceived risk. *Prenat Diagn* 2015;35:777–82.

63 Sirota M, Juanchich M, Kostopoulou O, *et al.* Decisive evidence on a smaller-than-you-think phenomenon: revisiting the "1-in-X" effect on subjective medical probabilities. *Med Decis Making* 2014;34:419–429.

64 Sirota M, Juanchich M, Petrova D, *et al.* Health professionals prefer to communicate risk-related numerical information using "1-in-X" ratios. *Med Decis Making* 2018;38:366–376.

65 Akl EA, Oxman AD, Herrin J, *et al.* Using alternative statistical formats for presenting risks and risk reductions. *Cochrane Database Syst Rev* 2011;3:CD006776.

66 Covey J. A meta-analysis of the effects of presenting treatment benefits in different formats. *Med Decis Making* 2007;27:638–654.

67 Gigerenzer G, Edwards A. Simple tools for understanding risks: From innumeracy to insight. *Br Med J* 2003;327:741.

68 Okan Y, Garcia-Retamero R, Cokely ET, *et al.* Individual differences in graph literacy: Overcoming denominator neglect in risk comprehension. *J Behav Decis Mak* 2012;25:390–401.

69 Paling J. Strategies to help patients understand risks. *Br Med J* 2003;327:745–748.

70 Schapira MM, Nattinger AB, McHorney CA. Frequency or probability? A qualitative study of risk communication formats used in health care. *Med Decis Making* 2001;21:459–467.

For peer review only

Figure captions

Figure 1. Flowchart representing the results of the website selection after applying exclusion criteria.

Figure 2. Results of content and format analysis for information concerning cervical screening (a) benefits, (b) risks, (c) outcomes, and (d) cervical cancer statistics. A total of 14 websites were analyzed. Items marked with an asterisk were added by the authors to the checklist by Kolthoff et al. [28]. Details concerning instances in which multiple formats were used for a given information item can be found in online Supplementary materials (Table S3).

For peer review only

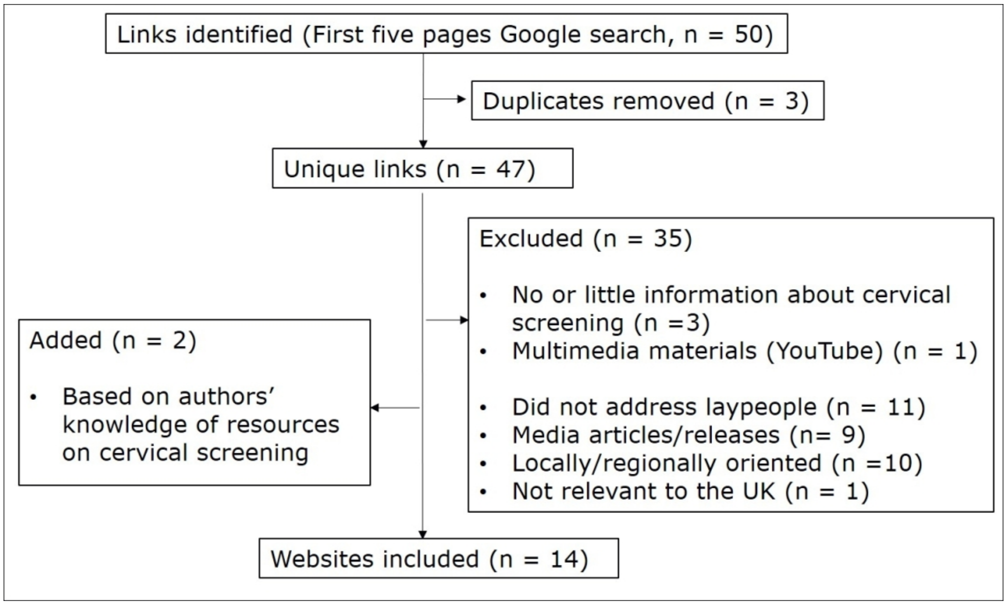

Figure 1. Flowchart representing the results of the website selection after applying exclusion criteria.

241x144mm (150 x 150 DPI)

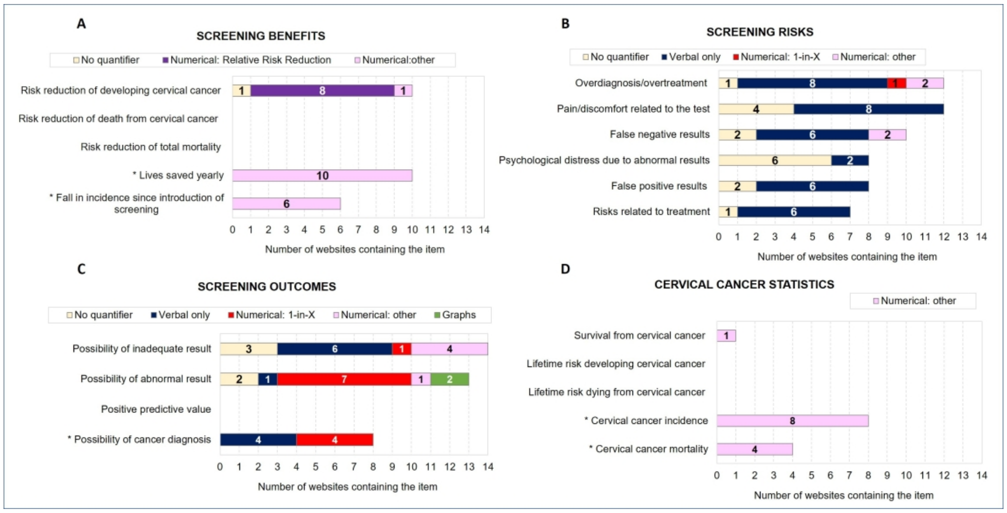

Figure 2. Results of content and format analysis for information concerning cervical screening (a) benefits, (b) risks, (c) outcomes, and (d) cervical cancer statistics. A total of 14 websites were analyzed. Items marked with an asterisk were added by the authors to the checklist by Kolthoff et al.[28]. Details concerning instances in which multiple formats were used for a given information item can be found in online Supplementary materials (Table S3).

550x278mm (150 x 150 DPI)

Supplementary materials for "How is cervical screening communicated in UK websites?"

Table S1. Websites coded and date of access for analysis.

Organization	Link	Date
British Society for Colposcopy and Cervical Pathology	https://www.bsccp.org.uk/	28/04/2017
BootsWebMD [a]	https://www.webmd.boots.com/	09/01/2018
Bupa UK	https:/www.bupa.co.uk	27/04/2017
Cancer Research UK	https://www.cancerresearchuk.org	27/04/2017
Jo's Cervical Cancer Trust [a]	https://www.jostrust.org.uk	27/04/2017
LGBT Foundation [b]	http://lgbt.foundation	27/04/2017
MacMillan Cancer Support [a]	https://www.macmillan.org.uk	27/04/2017
Marie Stopes UK [b]	https://www.mariestopes.org.uk	27/04/2017
NHS Choices (England) [a]	http://www.nhs.uk	20/03/2017
NHS Inform (Scotland) [a]	https://www.nhsinform.scot	20/03/2017
Patient Info	https://patient.info	27/04/2017
Public Health Agency Northern Ireland [a]	http://www.cancerscreening.hscni.net	26/04/2017
Public Health Wales [a]	http://www.cervicalscreeningwales.wales.nhs.uk	20/04/2017
Women's Health Concern	https://www.womens-health-concern.org	28/04/2017

Note: [a] Electronic leaflets and/or factsheets for the general public were available to download via a link this website, and were thus considered part of this resource; [b] Only an electronic leaflet was available and thus the analysis focuses on this resource.

For peer review only

Table S2. Codes from Kolthoff et al.'s (2016) checklist, with examples of corresponding statements. Codes marked with an asterisk were added by the authors.

**Screening benefits**	
Risk reduction of developing cervical cancer	Regular screening can cut the risk of getting cervical cancer by 75%.
	Cervical screening prevents 8 out of 10 cervical cancers from developing.
Risk reduction of death from cervical cancer	No statements found
Risk reduction of total mortality	No statements found
* Lives saved yearly	Cervical screening saves around 5,000 lives every year in the UK.
	Over 1000 lives are saved every year through regular screening.
* Fall in incidence since introduction of screening	The number of women who develop cervical cancer has halved since the 1980s due to most women regularly having cervical screening.
	Since the screening programme was introduced in the 1980s, the number of cervical cancer cases has decreased by about 7% each year.
**Screening risks**	
Overdiagnosis and overtreatment	The test can pick up minor abnormalities in cervical cells which could clear up by themselves.
	Your screening test may show up mild cell changes in your cervix, which would never have gone on to become cancer or caused you any problems. If this happens, you may receive treatment that you don't really need.
Pain/discomfort related to the test	Cervical screening tests are not painful, although some women find the speculum uncomfortable.
	You might feel some discomfort or pain - try to relax by taking slow, deep breaths as it may hurt more if you are tense.
False negative results	No screening test is 100% accurate. Some tests will be falsely reassuring (so-called false negative results) - where the test is reported as normal but an abnormality is present.
	Regular screening detects up to 80% of cervical abnormalities.
Psychological distress due to abnormal results	A diagnosis of and treatment for cervical abnormalities can cause significant anxiety for many women.

For peer review only

	Many women worry when an abnormality is found.
False positive results	False positive results might wrongly show changes in your cervix.
	Very occasionally, screening can show you have abnormal cells when in fact there's no problem. This is called a false positive result.
Risks related to treatment	For a few women, the treatments may cause problems such as bleeding afterwards or a small increase in future pregnancies of having the baby early.
	Very rarely, the cervix can become tightly closed after treatment. This known as stenosis. It can make it harder for the sperm to enter the womb and so can affect your chances of becoming pregnant naturally.
**Screening outcomes**	
Possibility of inadequate result	Sometimes test results are declared 'unsatisfactory' because the slide was poorly prepared or difficult to read. In such cases, the test needs to be repeated and the patient will be called back.
	An inadequate result does not mean your smear was abnormal but that it was unreadable. This means that there were not enough cells in the sample and happens in about 3 out of every 100 tests taken.
Possibility of abnormal result	Most abnormal results from screening tests show only very minor changes.
	About 1 in 20 women will have a smear test result which is abnormal.
Positive predictive value	No statements found
* Possibility of cancer diagnosis	A cervical screening test can very occasionally find early cervical cancer. Most women with an abnormal test result have early cell changes and not cancer.
	It is extremely rare for cancer to be diagnosed from a cervical screening test. Less than one in 1,000 women's test results show invasive cancer.
**Cervical cancer statistics**	
Survival from cervical cancer	Overall, almost 2 out of 3 women (63%) survive ten years or more, and more than 8 out of 10 women (83%) will live at least one year.
Lifetime risk of developing cervical cancer	No statements found

For peer review only

Lifetime risk of dying from cervical cancer	No statements found
* Cervical cancer incidence	Around 3,000 women are diagnosed with cancer of the cervix every year.
	Each year in Northern Ireland, about 103 women are diagnosed with cervical cancer.
* Cervical cancer mortality	Sadly around 900 women die of cervical cancer each year in England.
	In Northern Ireland, 20–30 women die each year from cervical cancer.

For peer review only

Table S3a. Information items about cervical screening benefits, risks, outcomes, and cervical cancer statistics (Part 1)

	BSCCP	Boots WebMD	Bupa UK	Cancer Research UK	Jo's Cervical Cancer Trust	LGBT Foundation	MacMillan Cancer Support
**Screening benefits**							
Risk reduction of developing cervical cancer	-	-	-	RR	RR	RR	0
Risk reduction of death from cervical cancer	-	-	-	-	-	-	-
Risk reduction of total mortality	-	-	-	-	-	-	-
* Lives saved yearly	-	-	N	N	N	-	N
* Fall in incidence since the introduction of screening [a]	N	-	-	N	-	-	N
**Screening risks**							
Overdiagnosis and overtreatment	V	-	V	V	V, N	V	V
Pain/discomfort related to the test	-	0	0	0	V	V	V
False negative results	-	-	0	0	V, N	-	V
Psychological distress due to abnormal results	0	-	0	0	V	V	0
False positive results	V	-	V	V	-	-	V
Risks related to treatment	V	-	-	V	V	-	V
**Screening outcomes**							
Possibility of inadequate result	0	V	V, N	V	V	0	V
Possibility of abnormal result	1X, N	0	1X	1X, N	V, 1X	V, 1X	V
Positive predictive value	-	-	-	-	-	-	-
* Possibility of cancer diagnosis	V, 1X, N	-	-	V	V, 1X	V	V
**Cervical cancer statistics**							
Survival from cervical cancer	-	N	-	-	-	-	-
Lifetime risk of developing cervical cancer	-	-	-	-	-	-	-
Lifetime risk of dying from cervical cancer	-	-	-	-	-	-	-
Cervical cancer incidence	-	N	N	-	N	-	-
Cervical cancer mortality	-	-	-	-	N	N	-
**Tone**							
Appeal for participation	x	x	x	x	x	x	x
Informed decision making	-	-	x	-	x	-	-

Note: - = item not present; 0 = not quantified; V = verbal quantifier; RR = numerical relative risk reduction; 1X = numerical 1-in-X; N = numerical (other); G = graph; x = item present (coding format not applicable); * codes added by the authors; [a] includes estimates of incidence at different time points, as well as any mention to the magnitude of the fall in incidence (e.g., rates have halved).

For peer review only

Table S3b. Information items about cervical screening benefits, risks, outcomes, and cervical cancer statistics (Part 2)

	Marie Stopes UK	NHS Choices (England)	NHS Inform (Scotland)	Patient Info	PHA Northern Ireland	Public Health Wales	Women's Health Concern
**Screening benefits**							
Risk reduction of developing cervical cancer	RR	N	RR	RR	RR	RR	-
Risk reduction of death from cervical cancer	-	-	-	-	-	-	-
Risk reduction of total mortality	-	-	-	-	-	-	-
* Lives saved yearly	N	N	N	N	-	N	N
* Fall in incidence since the introduction of screening [a]	-	N	-	N	-	N	-
**Screening risks**							
Overdiagnosis and overtreatment	0	V	V	V, N	-	V, 1X	V
Pain/discomfort related to the test	V	V	-	V	V	V	0
False negative results	-	V	V	V	V	V	V, N
Psychological distress due to abnormal results	-	0	-	0	-	-	-
False positive results	-	V	0	0	-	V	-
Risks related to treatment	-	V	-	-	0	V	-
**Screening outcomes**							
Possibility of inadequate result	0	V, N	V	N	V, N	1X, N	V
Possibility of abnormal result	0	V, 1X, N, G	V, 1X, G	V, N	V, 1X, N	V, 1X, N	-
Positive predictive value	-	-	-	-	-	-	-
* Possibility of cancer diagnosis	-	V, 1X	-	V, 1X	-	V	-
**Cervical cancer statistics**							
Survival from cervical cancer	-	-	-	-	-	-	-
Lifetime risk of developing cervical cancer	-	-	-	-	-	-	-
Lifetime risk of dying from cervical cancer	-	-	-	-	-	-	-
Cervical cancer incidence	N	N	-	N	N	N	-
Cervical cancer mortality	-	-	-	-	N	N	-
**Tone**							
Appeal for participation	-	-	x	x	x	x	x
Informed decision making	-	x	x	-	-	x	x

Note: - = item not present; 0 = not quantified; V = verbal quantifier; RR = numerical relative risk reduction; 1X = numerical 1-in-X; N = numerical (other); G = graph; x = item present (coding format not applicable); * codes added by the authors; [a] includes estimates of incidence at different time points, as well as any mention to the magnitude of the fall in incidence (e.g., rates have halved).

For peer review only

BMJ Open

How is cervical cancer screening information communicated in UK websites? A cross-sectional analysis of content and quantitative presentation formats

Journal:	*BMJ Open*
Manuscript ID	bmjopen-2019-029551.R1
Article Type:	Original research
Date Submitted by the Author:	05-Aug-2019
Complete List of Authors:	Okan, Yasmina; University of Leeds, Centre for Decision Research, Leeds University Business School<br>Smith, Samuel; University of Leeds, Leeds Institute of Health Sciences<br>Bruine de Bruin, Wändi; University of Leeds, Centre for Decision Research, Leeds University Business School; Carnegie Mellon University, Department of Engineering and Public Policy
**Primary Subject Heading**:	Communication
Secondary Subject Heading:	Public health
Keywords:	cervical cancer screening, informed decision making, risk communication, risk perception, web analysis

SCHOLARONE™
Manuscripts

Running Head: CERVICAL CANCER SCREENING WEBSITES

Word count: 3,917

How is cervical cancer screening information communicated in UK websites? A cross-sectional analysis of content and quantitative presentation formats

Yasmina Okan,[1 *] Samuel G. Smith,[2] Wändi Bruine de Bruin,[1,3]

[1]Centre for Decision Research, Leeds University Business School, University of Leeds, Leeds, UK

[2] Leeds Institute of Health Sciences, University of Leeds, Leeds, UK

[3]Department of Engineering and Public Policy, Carnegie Mellon University, Pittsburgh, US

* Corresponding author at Centre for Decision Research, Leeds University Business School, University of Leeds, Charles Thackrah Building, Leeds, LS2 9LB, UK; E-mail: y.okan@leeds.ac.uk, Phone: +44 113 343 2622.

For peer review only

Abstract

Objectives: To investigate whether UK websites about cervical cancer screening targeted to the public include (1) information about benefits and risks of screening, possible screening results, and cervical cancer statistics, (2) quantitative presentation formats recommended in the risk communication literature, and (3) appeals for participation and/or informed decision making.

Design: Cross-sectional analysis of websites using a comprehensive checklist containing information items on screening benefits, risks, possible results, and cervical cancer statistics.

Outcome measures: We recorded the number of websites that contained each of the information items, and the presentation format used for probabilistic information (no quantification provided, verbal quantifiers only, different types of numerical formats, and/or graphs). We also recorded the number of websites containing appeals for participation and/or references to informed decision making.

Setting: Websites were identified through the most common Google search terms used in the UK to find information on cervical screening, according to GoogleTrends and a commercial internet-monitoring programme. Two additional websites were identified by the authors as relevant.

Results: After applying exclusion criteria, 14 websites were evaluated, including websites of public and private health service providers, charities, a medical society, and a pharmacy. Content of the websites included different benefits, risks of screening, and possible results. However, specific content varied between websites. Probabilistic information was often presented using non-recommended formats, including relative risk reductions to express screening benefits, and verbal quantifiers

For peer review only

without numbers to express risks. Appeals for participation without mention of informed decision making occurred in half of the websites.

Conclusions: UK websites about cervical cancer screening may not fully support informed decision making about screening participation. Although websites were generally balanced, the specific content varied, and recommendations from the literature to facilitate understanding of quantitative information were often not followed. Designing websites that adhere to existing recommendations may support informed screening uptake.

Keywords: cervical cancer screening; informed decision making; risk communication; risk perception; web analysis

For peer review only

Article summary

Strengths and limitations of this study

- We analyzed the content of UK websites about cervical cancer screening using an established checklist of information items, and identified additional information that was mentioned commonly.
- We systematically examined whether cancer screening websites present probabilistic information in formats recommended in the risk communication literature.
- We identified websites by applying the most common Google search terms used in the UK to find information on cervical screening, and examined the majority of links that users will realistically access.
- All the information items we assessed may not be relevant for screening decisions, whereas we may have omitted some information that can be relevant (e.g., about the human papillomavirus).
- We did not examine whether websites mentioned the uncertainty associated with estimates of benefits and risks, although such information can be important for informed decision making.

For peer review only

INTRODUCTION

Cervical cancer is highly preventable. It is caused in most cases by an infection with the human papillomavirus (HPV), which may lead to abnormal changes in cervical cells[1,2]. Such abnormalities can be detected through screening and treated before they become cancerous[3]. Indeed, cervical screening significantly reduces both cervical cancer incidence and cancer-specific mortality[3–7]. In the UK, age-standardized incidence was 11.8 in 100,000 in 2014, and age-standardized mortality was 3.3 in 100,000[8]. In England alone, estimates suggest that there would be 1,827 additional cervical cancer deaths per year without screening[7].

However, cervical screening is associated with potential risks. These include detection and treatment of abnormal cells that would have cleared up on their own (i.e., overdiagnosis and overtreatment[9,10]), and increasing risk of preterm birth as relatively larger amounts of tissue are removed during treatment[11–13]. A recent meta-analysis indicates that about half of untreated moderately abnormal cells regress within two years[9], implying the possibility of overtreatment. However, experts and policy makers generally agree that benefits of cervical screening outweigh potential risks[5,14,15]. In the UK, the National Health Service (NHS) offers screening to all women aged 25-64. It has been emphasized that screening invitees need information about both benefits and risks to make informed decisions about participation[14,16,17].

Organized screening programs often use written outreach materials. In the UK, eligible women receive an invitation letter with an information leaflet that mentions websites about cervical screening. Hence it is important to examine whether widely accessed websites effectively support decisions about screening participation. Both European and UK guidelines have emphasized that cancer screening

communications should be comprehensive in content, and should provide balanced discussions of benefits and risks to screening[17–20]. Such guidelines have also highlighted that screening communications should be comprehensible, and avoid quantitative presentations that are hard to understand[17–19]). Communications that are not well understood can cause undue concern, reduce recipients' self-efficacy beliefs about their capacity to participate in screening, and undermine informed uptake[21,22].

Quantitative information in screening communications, however, can be challenging even for educated audiences[21,23]. Such information can be presented using a range of formats, including verbal quantifiers (e.g., "low" or "moderate" risk), various numerical formats, and graphical displays (e.g., icon arrays). Research in risk communication has shown that quantitative presentation formats vary considerably in their effectiveness to promote accurate understanding. Some formats are known to be relatively ineffective, while others still lack conclusive evidence[24]. Table 1 provides an overview of quantitative formats, associated evidence-based recommendations and supporting references. Key recommendations relevant to screening communications include avoiding the use of verbal quantifiers without accompanying numbers; numerical "1-in-X" formats; and presentations of risk reductions in relative terms. Another recommendation is to add simple graphical displays to numerical information.

For peer review only

Table 1. Evidence-based recommendations from the risk communication literature to promote understanding of probabilistic information

Recommendation	Rationale	Key references (first author)
Avoid the use of verbal quantifiers without numbers (e.g., women who have abnormal cells removed are *slightly more likely* to have their baby early").	Interpretations of verbal quantifiers vary across individuals and often differ from interpretations intended by communicators. Verbal quantifiers can lead to overestimations of risks.	Budescu [25]; Knapp [26,27]; Peters [28]; Visschers et al. [29]; Young [30]; Zipkin [31]
Avoid numerical "1-in-X" formats to present the chance of an outcome (e.g., 1 in 12 women will have an abnormal test result).	People tend to perceive the same probabilities as higher and more worrying when presented in a "1-in-X" format, as compared to numerically equivalent "N-in-X*N" ratios (e.g., 10 in 120 women will have an abnormal test result) or percentages (e.g., 5% women will have an abnormal test result).	Pighin [32,33]; Sirota [34–36]; Trevena [37]; Zikmund-Fisher [38]
Avoid presenting estimates of risk reduction in relative terms (e.g. screening cuts the risk of getting cervical cancer by 75%).	Relative risk differences can obscure the true magnitude of benefit or harm, as compared to absolute risk differences (e.g., screening reduces the risk of getting cervical cancer from 20 in 1,000 to 5 in 1,000).	Akl [39]; Covey [40]; Fagerlin [22], Gigerenzer [21,41]; Trevena [37] Zipkin [31]

For peer review only

Add simple graphical displays of numerical information (e.g., icon arrays, where icons of different colors represent those affected and not affected by the risk).	Well-designed, simple graphs help to overcome difficulties in understanding numerical information and are often perceived as more appealing and easier to understand.	Galesic [42]; Garcia-Retamero [43]; Gigerenzer [41]; Okan [44]; Paling [45]; Schapira [46]

For peer review only

Existing guidelines also state that screening communications should not persuade people to attend or present screening as necessary or important, without acknowledging that not participating is a reasonable choice[17–20]. Recent guidance from the UK National Screening Committee[17] notes that “information should make it clear that it is a personal choice to accept or decline screening and both choices will be fully supported” (p. 6). This approach to screening communications seeks to respect personal autonomy and ensure that decisions are in line with invitees’ personal values and circumstances – especially since healthy individuals can be adversely affected by screening[47,48]. Besides undermining the principle of autonomy, persuasive messages may have unintended negative effects, such as eliciting guilt and anxiety among invitees who decline the offer, anger among those who participate and are harmed as a result, and potential mistrust in communicators over time[20,48,49].

We investigated whether UK websites about cervical screening adhere to existing guidelines and policy recommendations concerning information content and appeals for informed decision making (vs. for participation), as well as to recommendations from the risk communication literature concerning quantitative presentation formats. Previous website analyses have primarily focused on breast cancer screening, and have generally assessed only website content[47,50], or a specific aspect of presentation format (e.g., consistency in the presentation of statistics on overdiagnosis[51]).

We used an established checklist of information items about cervical screening[52], building on earlier evaluations of breast cancer screening communications[47,53,54]. We assessed quantitative presentation formats

For peer review only

considering the recommendations listed in Table 1. Additionally, we examined the type of appeals included in websites. In sum, our research questions were:

1. Do UK websites about cervical screening contain a) key information about screening benefits and risks, possible results, and b) cervical cancer statistics?
2. Do UK websites about cervical screening present probabilistic information using recommended formats?
3. Do UK websites about cervical screening contain appeals for participation and/or informed decision making?

METHODS

Search strategy

We used GoogleTrends to identify the most common Google search terms used in the UK to find information on cervical screening. We identified search terms related to "cervical screening" in the 12 months prior to March 2017. We used "cervical screening" because it is used in the screening invitations from the UK's National Health Service. Related search terms included "cervical cancer screening," "cervical smear," and "smear test." To identify which of these terms was more common, we used Wordtracker (https://app.wordtracker.com/), a commercial program that estimates the relative frequency of Google search terms in a given period of time (see[55]for a similar procedure). The two most commonly used terms in the UK in the year before March 2017 were "smear test" and "cervical screening."

Website selection

On March 9th, 2017, we performed the Google search '"smear test" OR "cervical screening"' using the private Firefox browsing mode to prevent previously visited pages and cookies from influencing search results. Following Ghanouni et al.[51], we examined the first five pages of results using the default of 10 results per page,

For peer review only - http://bmjopen.bmj.com/site/about/guidelines.xhtml

resulting in 50 links. This includes the vast majority of websites that users will access (see also[56]). The full list of links is available at the Open Science Framework (https://osf.io/73gfn/?view_only=1dbf5d9e16c64b2aaa2cd3af704e7103)

We excluded links that (1) targeted healthcare professionals and academics rather than laypeople (e.g., technical reports, research articles), (2) reflected online media articles or press releases, (3) presented international information not applicable to the UK (e.g., Wikipedia's overview of screening recommendations in different countries), and (4) were locally or regionally-oriented. The latter links typically focused on basic practical aspects (e.g., who is eligible for screening and logistics of making appointments for specific GP surgeries), and often included links for further information to national websites included in our analyses. We also excluded links that (5) were duplicate, (6) included no or little information about cervical screening (<150 words), (7) included no written materials (e.g., YouTube video). Additionally, we added two websites identified through our knowledge of resources on cervical screening (Patient and Women's Health Concern). Although these websites did not appear in the Google search, they represent trusted UK resources that some women may access directly to learn about cervical screening. Figure 1 summarizes the results of the website selection, discussed in detail below.

For the websites identified through Google, we coded the link listed in the search results. For the two additional websites, we coded the main link providing information about cervical screening. In all cases, we also coded sections on cervical screening within each website that could be directly accessed from the initial link, as well as other written materials directly accessible, including electronic leaflets, fact sheets, and slideshows, which were all considered as part of the same resource in analyses. Websites were accessed between 20th March 2017 and 28th April 2017, except one

website (BootsWebMD), which was accessed at a later date due to an oversight following the initial website selection. The date of accessing each website, corresponding links, and estimated number of visits appears in Supplementary materials (Table S1). Copies of PDF files reflecting the content of all websites at the time of access are available at the Open Science Framework.

Coding of websites

To code website content, we adapted a checklist of information items that was developed to analyze invitations for cervical screening[52]. Following European and UK guidelines[18,19], the checklist included items about screening benefits, risks, and possible results (e.g., the possibility of an abnormal or an inadequate result). Following Kolthoff et al.[52] the item on overdiagnosis/overtreatment included any reference to screening possibly detecting abnormal cells that may clear up on their own and/or leading to unnecessary treatment, regardless of whether the treatment type was mentioned (see also[57,58]). The checklist also included items about cervical cancer statistics (e.g., lifetime risk of developing cervical), which may be relevant for screening decisions[47,53,54]. After initial inspection of the websites, we added five items to the checklist to reflect commonly presented information that may also influence screening decisions (Figure 2). Examples of all information items appear in Supplementary materials (Table S2).

To assess quantitative presentation formats, we coded whether probabilistic information was presented using: no quantifiers, verbal quantifiers only, numerical quantifiers (1-in-X vs. other numerical format), and/or graphs. We also coded whether any information about risk reduction was presented in relative terms. Multiple formats (e.g., graphs accompanied by numbers) were present in some communication materials (Table S3, Supplementary materials).

For peer review only

Finally, we coded appeals for informed decision making (e.g., “deciding whether to have screening or not is your choice”) and appeals for participation. Following Kotlhoff et al.[52], the latter included direct encouragement for participation (e.g., “a smear test—have it”) and statements presenting screening as necessary (e.g., “it’s essential for women to have regular cervical screening tests”) or important (e.g., “it is important to go for screening every time you are invited”).

The first author (YO) read through all selected materials and applied the coding scheme. Three websites (>20%) were coded by another author (SS). Cohen’s kappa was moderate to high on all codes except ‘article tone’ (.18). Discrepancies for this code were caused by appeals for participation that were not prominent. These discrepancies were resolved through discussion, resulting in consensus. The average Cohen’s kappa was .86 (range: .65 to 1).

Patient and public involvement

Patients and/or public were not involved in the design or conduct of the study, which did not involve human participants.

RESULTS

Website selection

As described above, we identified 50 links through our Google search. After exclusions, 12 websites remained (Figure 1), including 4 websites from public health service providers in the UK’s four countries (NHS Choices England, NHS Inform Scotland, Public Health Agency Northern Ireland, Public Health Wales), one private health service provider (Bupa UK), three cancer charities (Cancer Research UK, Jo’s Cervical Cancer Trust, MacMillan Cancer Support), two additional charities (LGBT Foundation, Marie Stopes UK), one medical society (British Society for Colposcopy and Cervical Pathology), and one pharmacy (Boots WebMD). All websites from

For peer review only

public health service providers included links to official invitation leaflets, which were coded with the corresponding website, as described above. As noted earlier, two additional websites (Patient and Women's Health Concern) were identified by the authors. Below, we present summary statistics across websites. Results for individual websites appear in online Supplementary materials (Table S3).

<Figure 1>

Do UK websites about cervical screening contain a) key information about screening benefits and risks, possible results, and b) cervical cancer statistics?

We report on four main findings regarding website content. First, screening benefits were mentioned frequently, although the focus was primarily on risk reduction of developing cervical cancer (Figure 2a). Estimates of risk reduction typically varied between 70% and 80%. Based on Sasieni et al.[59], two websites provided an estimated range of 60-80%, and another provided an estimate of 90% for the reduction in the risk of advanced cancer specifically. No websites explicitly mentioned risk reductions of death from cervical cancer or total mortality. Instead, benefits were often expressed in terms of the number of lives saved yearly (e.g. "cervical screening saves 5,000 lives a year in the UK"; n=10 websites), or in some cases referring to the fall in cervical cancer cases since the national screening programme was initiated (e.g., "since the screening programme was introduced in the 1980s, the number of cervical cancer cases has decreased by about 7% each year"; n=6).

Second, screening risks were also mentioned relatively frequently (Figure 2b), but the specific risks and their descriptions varied across websites. The most commonly mentioned risks were overdiagnosis and overtreatment (n=12),

For peer review only

pain/discomfort related to the cytology test (n=12), and the possibility of false negatives (n=10). Descriptions of overdiagnosis and overtreatment, however, generally focused on cell changes often clearing up and not progressing into cancer (e.g., “in most cases, the abnormal cells will disappear on their own”). The risk of unnecessary additional tests or treatment was only mentioned explicitly in seven cases, often in connection to justifications for current screening age ranges (e.g., “changes in a young woman’s cervix are quite normal. In this situation, screening may lead to unnecessary treatment”). Most websites described the test as uncomfortable but not painful (e.g. “having the speculum put in may be a little uncomfortable, but it shouldn’t hurt”), though one website did state that the test was potentially painful. Risks related to treatment were only mentioned in half of the websites (Figure 2b), all of which mentioned the possibility of premature birth. Two websites also noted the risk of stenosis (i.e., the cervix becoming tightly closed after treatment).

Third, possible screening results were mentioned in most websites (Figure 2c). Estimates of the likelihood of inadequate results varied between 1.7 and 3% and estimates of the likelihood of abnormal results ranged between 5 and 10%. Additionally, the possibility of cancer diagnosis was mentioned in over half of the websites (n=8) and was often reported as occurring in less than 1 in 1,000 cases.

Finally, cervical cancer statistics such as lifetime risk of developing or dying from cervical cancer were seldom discussed, although some websites did provide details on cervical cancer incidence and mortality (Figure 2d). Specific estimates of incidence varied across websites, and included yearly, daily, national, and regional estimates (e.g., “each year in Northern Ireland, about 103 women are diagnosed with cervical cancer”).

For peer review only

<Figures 2a, 2b, 2c, and 2d>

Do UK websites about cervical screening present probabilistic information using recommended formats?

Information was often not presented in recommended formats. First, the recommendation to avoid the use of verbal quantifiers without numbers[31] was often not followed for information about risks (Figure 2b). Risks related to treatment were quantified only verbally, often using varying verbal quantifiers. For example, one website stated that “women are *slightly more likely* to have their baby 1 to 2 months early,” and another stated that “women may have a *higher risk* of preterm delivery.” Other risks were also either only quantified verbally or not quantified at all, with the exception of overdiagnosis/overtreatment (n=3) and false negative results (n=2).

Second, the recommendation to avoid “1-in-X” numerical formats[38] was often violated for information about screening results. This was the most popular format for conveying the likelihood of abnormal results (Figure 2c). Third, the recommendation to avoid presentations of relative risk reduction[21] was violated in most cases in which an estimate of risk reduction was provided (Figure 2a). Finally, the recommendation to add simple graphical displays[43] was often not met. Only two websites contained graphs, each depicting the likelihood of abnormal results.

Do UK websites about cervical screening contain appeals for participation and/or informed decision making?

Appeals for participation were present in most websites (n=12). Specifically, appeals for participation without mention of informed decision making occurred in half of the websites (n=7). Several websites combined appeals for participation and informed decision making (n=5) (e.g., “don’t ignore your smear test, it could save

For peer review only

your life" and "taking part in cervical screening is your choice"). One website (NHS Choices England) referred to informed decision making only ("deciding whether or not to have a cervical screening test is your choice"). The websites of the three remaining public health service providers included either both appeals for participation and informed decision making (NHS Inform Scotland and Public Health Wales) or only appeals for participation (Public Health Agency Northern Ireland).

DISCUSSION

We investigated whether UK websites about cervical screening adhered to existing recommendations about content, quantitative presentation formats, and type of appeals. We found that websites often followed recommendations from European and UK guidelines to include information about both benefits and risks of screening[17–19]. However, specific content varied across websites. For example, only half of the websites explicitly referred to risks related to the treatment of abnormal cells. Additionally, probabilistic information was often presented in formats not recommended in the risk communication literature, including relative risk reductions to express screening benefits, and verbal quantifiers without numbers to express risks. Moreover, several websites encouraged screening participation without referring to informed decision making, contrasting with current UK policy[14,60].

Our finding that websites often included information about both benefits and risks of cervical screening contrasts with previous reports that breast screening websites lacked information about risks such as overdiagnosis[47,50]. Recent emphasis in the UK on facilitating informed screening decisions[14,60] may have led to more comprehensive and balanced websites in recent years (see also[51]). The presence of information about both screening benefits and risks is aligned with women's

For peer review only

preferences, who often want to receive information on both aspects before their test[61]. However, our finding that specific content varied across websites suggests that women accessing different resources may come to different conclusions.

Our findings also suggest that existing descriptions of benefits and risks may not always support understanding. For example, the concepts of overdiagnosis and overtreatment are often unfamiliar and counterintuitive to people[62–64], highlighting the need to provide explanations that are accessible and transparent. Many websites did explain that cell changes often clear up on their own, potentially helping women to understand that cervical screening often finds indolent abnormalities (leading to unnecessary anxiety or worries[58]), but not necessarily that it can result in unnecessary treatment. Moreover, the websites that explicitly mentioned overtreatment often did so in relation to women below the recommended screening age. As a result, those within the recommended age may incorrectly infer that such risk does not apply to them. We also found that many websites emphasized that screening “saves lives.” It has been argued that such claims are misleading, and that communications should report reductions in cancer-specific mortality, overall mortality, as well as overall cancer deaths[65]. Although there is evidence that cervical screening reduces cancer-specific mortality[3–7], determining its impact on overall mortality requires large trials with sufficient power to detect differences[66].

Our finding that probabilistic information was often presented in non-recommended formats is concerning as this may cause misperceptions, even among educated audiences. The use of relative risk reductions to express screening benefits can hinder understanding and increase risk perceptions, relative to presentations of absolute risk reduction[31,37]. Although information about baseline risk (i.e., the absolute risk without screening) could reduce the biasing effect of

For peer review only

relative risks[67] (but see [68]), such beneficial effects may be limited to baseline risks presented in frequency formats (vs. probabilities)[69,70] and to more (vs. less) numerate recipients[70]. Moreover, we did not find such information in any of the websites. It has been argued, however, that presentations of relative risks may be considered for low-probability risks with high impact (e.g., an earthquake), to prevent people from neglecting such risks altogether[24,29].

Additionally, our results suggest that women may overestimate the likelihood of abnormal screening results due to the use of "1-in-X" formats (e.g., "1 in 12 women will have an abnormal test result")[36]. The use of verbal quantifiers without numbers to express cervical screening risks may also lead women to overestimate such risks [26–28,30]. The absence of numerical information can also lead people to perceive communications as less credible and trustworthy[29,71,72]. Although some risks are hard to quantify (e.g., psychological distress), estimates are available for risks such as preterm birth or overtreatment, from observational studies and meta-analyses[9,11,13]. The best available evidence could be presented in transparent fact boxes—a tabular format that facilitates comparisons of outcomes in groups of screened vs. unscreened individuals[73]. Fact boxes can also include simple graphs such as icon arrays to allow visual comparison of quantities[74], which could be especially beneficial for people with low numeracy. Yet, we found that graphs are seldom used in cervical screening websites.

Finally, our finding that some websites encouraged screening without mentioning informed decision making contrasts with current UK policy, which emphasizes that communications can recommend screening, but should acknowledge that not accepting the offer is a reasonable choice[14,20,60]. Although there is largely agreement that benefits of cervical screening outweigh potential risks[5,14,15],

For peer review only

persuasive messages raise ethical concerns to the extent that harms are possible[18,48,75]. Persuasive messages may also contribute to widespread enthusiasm for cancer screening (e.g.,[76,77]), discouraging people from reasoning about their screening choices[48]. Moreover, while guidelines for screening communications tend to focus on health service providers[17–19], conflicting recommendations from different sources might create confusion, negative beliefs about recommendations and scientific research, and potentially reduce screening intentions[78,79].

Strengths of our analyses include their comprehensiveness and systematic examination of website content using an established checklist of information items[52], and recommendations from the literature for quantitative presentation formats. Additionally, our analyses included most of the websites that UK citizens may realistically access, as our strategy for website selection was based on the top results identified by the most commonly used Google search terms. Our approach could be used by researchers and practitioners to evaluate the content and format of websites about related topics (e.g., other types of screening) in different countries. This would allow comparisons of websites for countries that have organized vs. opportunistic screening. For example, website content may be more heterogeneous in countries such as the United States, which do not have organized screening. It would also be interesting to compare different platforms (e.g., desktop websites vs. mobile tailored versions), and to examine whether website design (e.g., information position[80]) optimizes readability.

Limitations of our study include that our analyses may not have covered all information that is potentially relevant for screening decisions. For example, we did not assess whether websites mentioned information about the human papillomavirus

For peer review only

(HPV), or the uncertainty associated with estimated benefits and risks. Communicating uncertainty to laypeople can be challenging[81–83], but is important for informed decision making[16,17,60]. Conversely, it is possible that not all codes assessed are essential for informed screening decisions. We also did not directly assess whether users can make informed screening decisions based on the different websites. It is likely, however, that following guidelines and recommendations from the literature will facilitate better understanding and more informed decisions. Explicit appeals to informed decision making may also encourage users to evaluate the content, but of course do not guarantee that informed decisions will occur.

Future work should aim to identify essential information items for informed decisions about screening, considering both experts' and women's views. As noted by Ghanouni et al.[84], guidelines often do not provide detailed advice on *which* specific screening benefits, risks, or results should be discussed in communications. Moreover, experts may not fully agree on the relative importance of different information items, and women may have different information needs and preferences, including about whether and when to receive information about further tests. Providing too much unfamiliar information initially may also overwhelm screening invitees, and potentially distract them from key information necessary for decision making[22,85]. More research is also needed to identify how to best convey the individual significance of the population-level estimates included in screening communications, which may be hard for people to understand[86]. Finally, future studies should also examine the impact of different presentation formats on screening decisions(e.g., intentions to participate[87]) as evidence is still scarce. Such work would provide valuable insights to inform the design of evidence-based public communications about screening.

For peer review only

Acknowledgements

We are grateful to Mirta Galesic and Erika Waters for their valuable comments and suggestions.

Funding

This work was supported by a Population Research Fellowship awarded by Cancer Research UK to Yasmina Okan (reference C57775/A22182). Samuel G. Smith is supported by Yorkshire Cancer Research. Wändi Bruine de Bruin was partially supported by a grant from the Swedish Riksbanken Jubilieumsfond programme on Science and Proven Experience. The funding agencies had no involvement in designing the study, data collection, analysis and interpretation, writing the report, or the decision to submit the article for publication.

Competing interests

SS is an academic consultant for Luto, who are not involved in any of the websites reviewed in this manuscript.

Author contributions

YO conceived the research, acquired and analysed the data, and drafted the initial version of the manuscript. SS assisted with data analyses and interpretation. WBB contributed to data interpretation. All authors contributed to study design, revised the manuscript critically and approved the final version.

Data sharing statement

The full list of links initially identified and copies of PDF files reflecting the content of all analyzed websites at the time of access are available at the Open Science Framework (https://osf.io/73gfn/?view_only=1dbf5d9e16c64b2aaa2cd3af704e7103).

For peer review only

References

1 Crosbie EJ, Einstein MH, Franceschi S, *et al.* Human papillomavirus and cervical cancer. *The Lancet* 2013;382:889–99.

2 Muñoz N, Bosch FX, de Sanjosé S, *et al.* Epidemiologic classification of human papillomavirus types associated with cervical cancer. *N Engl J Med* 2003;348:518–27.

3 Peirson L, Fitzpatrick-Lewis D, Ciliska D, *et al.* Screening for cervical cancer: A systematic review and meta-analysis. *Syst Rev* 2013;2:35.

4 Nygård M. Screening for cervical cancer: when theory meets reality. *BMC Cancer* 2011;11:240.

5 Esserman LJ, Thompson IM, Reid B. Overdiagnosis and overtreatment in cancer: an opportunity for improvement. *J Am Med Assoc* 2013;310:797–798.

6 Peto J, Gilham C, Fletcher O, *et al.* The cervical cancer epidemic that screening has prevented in the UK. *Lancet* 2004;364:249–256.

7 Landy R, Pesola F, Castañón A, *et al.* Impact of cervical screening on cervical cancer mortality: Estimation using stage-specific results from a nested case–control study. *Br J Cancer* 2016;115:1140–1146.

8 Smittenaar CR, Petersen KA, Stewart K, *et al.* Cancer incidence and mortality projections in the UK until 2035. *Br J Cancer* 2016;115:1147–1155.

9 Tainio K, Athanasiou A, Tikkinen KA, *et al.* Clinical course of untreated cervical intraepithelial neoplasia grade 2 under active surveillance: Systematic review and meta-analysis. *Br Med J* 2018;360:k499.

10 McCredie MR, Sharples KJ, Paul C, *et al.* Natural history of cervical neoplasia and risk of invasive cancer in women with cervical intraepithelial neoplasia 3: A retrospective cohort study. *Lancet Oncol* 2008;9:425–434.

For peer review only

11 Castanon A, Landy R, Brocklehurst P, *et al.* Risk of preterm delivery with increasing depth of excision for cervical intraepithelial neoplasia in England: Nested case-control study. *Br Med J* 2014;349:g6223.

12 Sasieni P, Castanon A, Landy R, *et al.* Risk of preterm birth following surgical treatment for cervical disease: executive summary of a recent symposium. *BJOG Int J Obstet Gynaecol* 2016;123:1426–1429.

13 Kyrgiou M, Athanasiou A, Kalliala IEJ, *et al.* Obstetric outcomes after conservative treatment for cervical intraepithelial lesions and early invasive disease. *Cochrane Database Syst Rev* 2017;11:CD012847.

14 Forbes LJ, Ramirez AJ. Communicating the benefits and harms of cancer screening. *Curr Oncol Rep* 2014;16:382.

15 Moyer VA. Screening for cervical cancer: US Preventive Services Task Force recommendation statement. *Ann Intern Med* 2012;156:880–891.

16 Hersch JK, Nickel BL, Ghanouni A, *et al.* Improving communication about cancer screening: Moving towards informed decision making. *Public Health Res Pract* 2017;27:e2731728.

17 UK National Screening Committee. Guidance for the development, production and review of information to support UK population screening programmes. 2018. https://www.gov.uk/government/publications/uk-national-screening-committee-information-development-guidance (accessed 17 Aug 2018).

18 European Commission. *European guidelines for quality assurance in cervical cancer screening. 2nd edition.* Luxembourg: : Office for Official Publications of the European Communities 2008. http://screening.iarc.fr/doc/ND7007117ENC_002.pdf (accessed 17 Aug 2018).

For peer review only

19 General Medical Council. Consent: patients and doctors making decisions together. 2008. https://www.gmc-uk.org/ethical-guidance/ethical-guidance-for-doctors/consent (accessed 17 Aug 2018).

20 Entwistle VA, Carter S, Trevena L, *et al.* Communicating about screening. *Br Med J* 2008;333:789–91.

21 Gigerenzer G, Gaissmaier W, Kurz-Milcke E, *et al.* Helping doctors and patients make sense of health statistics. *Psychol Sci Public Interest* 2007;8:53–96.

22 Fagerlin A, Zikmund-Fisher BJ, Ubel PA. Helping patients decide: ten steps to better risk communication. *J Natl Cancer Inst* 2011;103:1436–1443.

23 Schwartz LM, Woloshin S, Black WC, *et al.* The role of numeracy in understanding the benefit of screening mammography. *Ann Intern Med* 1997;127:966–972.

24 Spiegelhalter D. Risk and uncertainty communication. *Annu Rev Stat Its Appl* 2017;4:31–60.

25 Budescu DV, Por H-H, Broomell SB, *et al.* The interpretation of IPCC probabilistic statements around the world. *Nat Clim Change* 2014;4:508–512.

26 Knapp P, Raynor DK, Berry DC. Comparison of two methods of presenting risk information to patients about the side effects of medicines. *BMJ Qual Saf* 2004;13:176–180.

27 Knapp P, Gardner PH, Woolf E. Combined verbal and numerical expressions increase perceived risk of medicine side-effects: A randomized controlled trial of EMA recommendations. *Health Expect* 2016;19:264–274.

28 Peters E, Hart PS, Tusler M, *et al.* Numbers matter to informed patient choices: A randomized design across age and numeracy levels. *Med Decis Making* 2014;34:430–442.

For peer review only

29 Visschers VH, Meertens RM, Passchier WW, *et al.* Probability information in risk communication: A review of the research literature. *Risk Anal* 2009;29:267–287.

30 Young S, Oppenheimer DM. Effect of communication strategy on personal risk perception and treatment adherence intentions. *Psychol Health Med* 2009;14:430–442.

31 Zipkin DA, Umscheid CA, Keating NL, *et al.* Evidence-based risk communication: a systematic review. *Ann Intern Med* 2014;161:270–280.

32 Pighin S, Savadori L, Barilli E, *et al.* The 1-in-X effect on the subjective assessment of medical probabilities. *Med Decis Making* 2011;31:721–729.

33 Pighin S, Savadori L, Barilli E, *et al.* Communicating Down syndrome risk according to maternal age: "1-in-X" effect on perceived risk. *Prenat Diagn* 2015;35:777–82.

34 Sirota M, Juanchich M, Kostopoulou O, *et al.* Decisive evidence on a smaller-than-you-think phenomenon: revisiting the "1-in-X" effect on subjective medical probabilities. *Med Decis Making* 2014;34:419–429.

35 Sirota M, Juanchich M, Petrova D, *et al.* Health professionals prefer to communicate risk-related numerical information using "1-in-X" ratios. *Med Decis Making* 2018;38:366–376.

36 Sirota M, Juanchich M, Bonnefon J-F. 1-in-X" bias: "1-in-X" format causes overestimation of health-related risks. *J Exp Psychol Appl* Published Online First: 2018.

37 Trevena LJ, Zikmund-Fisher BJ, Edwards A, *et al.* Presenting quantitative information about decision outcomes: a risk communication primer for patient decision aid developers. *BMC Med Inform Decis Mak* 2013;13:S7.

For peer review only

38 Zikmund-Fisher BJ. Continued use of 1-in-X risk communications is a systemic problem. *Med Decis Making* 2014;34:412–413.

39 Akl EA, Oxman AD, Herrin J, *et al.* Using alternative statistical formats for presenting risks and risk reductions. *Cochrane Database Syst Rev* 2011;3:CD006776.

40 Covey J. A meta-analysis of the effects of presenting treatment benefits in different formats. *Med Decis Making* 2007;27:638–654.

41 Gigerenzer G, Edwards A. Simple tools for understanding risks: From innumeracy to insight. *Br Med J* 2003;327:741−744.

42 Galesic M, Garcia-Retamero R, Gigerenzer G. Using icon arrays to communicate medical risks: Overcoming low numeracy. *Health Psychol* 2009;28:210–216.

43 Garcia-Retamero R, Cokely ET. Designing visual aids that promote risk literacy: A systematic review of health research and evidence-based design heuristics. *Hum Factors* 2017;59:582–627.

44 Okan Y, Garcia-Retamero R, Cokely ET, *et al.* Individual differences in graph literacy: Overcoming denominator neglect in risk comprehension. *J Behav Decis Mak* 2012;25:390–401.

45 Paling J. Strategies to help patients understand risks. *Br Med J* 2003;327:745–748.

46 Schapira MM, Nattinger AB, McHorney CA. Frequency or probability? A qualitative study of risk communication formats used in health care. *Med Decis Making* 2001;21:459–467.

47 Jørgensen KJ, Gøtzsche PC. Presentation on websites of possible benefits and harms from screening for breast cancer: Cross sectional study. *Br Med J* 2004;328:148.

For peer review only

48 Rychetnik L, Carter SM, Abelson J, *et al.* Enhancing citizen engagement in cancer screening through deliberative democracy. *J Natl Cancer Inst* 2013;105:380–386.

49 Raffle AE. Information about screening–is it to achieve high uptake or to ensure informed choice? *Health Expect* 2001;4:92–98.

50 Attena F, Cancellieri M, Pelullo CP. Scarce information about breast cancer screening: An Italian websites analysis. *Medicine (Baltimore)* 2016;95:e5615.

51 Ghanouni A, Meisel SF, Hersch J, *et al.* Information on “overdiagnosis” in breast cancer screening on prominent United Kingdom-and Australia-oriented health websites. *PLoS One* 2016;11:e0152279.

52 Kolthoff SK, Hestbech MS, Jørgensen KJ, *et al.* Do invitations for cervical screening provide sufficient information to enable informed choice? A cross-sectional study of invitations for publicly funded cervical screening. *J R Soc Med* 2016;109:274–281.

53 Slaytor EK, Ward JE. How risks of breast cancer and benefits of screening are communicated to women: Analysis of 58 pamphlets. *Br Med J* 1998;317:263–264.

54 Jørgensen KJ, Gøtzsche PC. Content of invitations for publicly funded screening mammography. *Br Med J* 2006;332:538–541.

55 McHugh SM, Corrigan M, Morney N, *et al.* A quantitative assessment of changing trends in internet usage for cancer information. *World J Surg* 2011;35:253–257.

56 Liebl P, Seilacher E, Koester M-J, *et al.* What cancer patients find in the internet: The visibility of evidence-based patient information - Analysis of information on German websites. *Oncol Res Treat* 2015;38:212–218.

For peer review only

57 Phillips K, Hersch J, Turner R, *et al.* The influence of the 'cancer effect'on young women's responses to overdiagnosis in cervical screening. *Patient Educ Couns* 2016;99:1568–1575.

58 Brodersen J, Schwartz LM, Heneghan C, *et al.* Overdiagnosis: what it is and what it isn't. *BMJ Evid-Based Med* 2018;23:1–3.

59 Sasieni P, Castanon A, Cuzick J, *et al.* Effectiveness of cervical screening with age: population based case-control study of prospectively recorded data. *Br Med J* 2009;339:b2968.

60 Forbes LJ, Ramirez A-J, list) E group on I about BS. Offering informed choice about breast screening. *J Med Screen* 2014;21:194–200.

61 Dieng M, Trevena L, Turner RM, *et al.* What Australian women want and when they want it: Cervical screening testing preferences, decision-making styles and information needs. *Health Expect* 2013;16:177–188.

62 Waller J, Whitaker KL, Winstanley K, *et al.* A survey study of women's responses to information about overdiagnosis in breast cancer screening in Britain. *Br J Cancer* 2014;111:1831–1835.

63 Nagler RH, Franklin Fowler E, Gollust SE. Women's awareness of and responses to messages about breast cancer overdiagnosis and overtreatment. *Med Care* 2017;55:879–885.

64 Ghanouni A, Renzi C, Waller J. Improving public understanding of 'overdiagnosis' in England: A population survey assessing familiarity with possible terms for labelling the concept and perceptions of appropriate terminology. *BMJ Open* 2018;8:e021260.

65 Gigerenzer G. Full disclosure about cancer screening. *Br Med J* 2016;352:h6967.

For peer review only

66 Prasad V, Lenzer J, Newman DH. Why cancer screening has never been shown to “save lives”—and what we can do about it. *Br Med J* 2016;352:h6080.

67 Natter HM, Berry DC. Effects of presenting the baseline risk when communicating absolute and relative risk reductions. *Psychol Health Med* 2005;10:326–334.

68 Sorensen L, Gyrd-Hansen D, Kristiansen IS, *et al.* Laypersons’ understanding of relative risk reductions: randomised cross-sectional study. *BMC Med Inform Decis Mak* 2008;8:31.

69 Covey J. The effects of absolute risks, relative risks, frequencies, and probabilities on decision quality. *J Health Commun* 2011;16:788–801.

70 Bodemer N, Meder B, Gigerenzer G. Communicating relative risk changes with baseline risk: Presentation format and numeracy matter. *Med Decis Making* 2014;34:615–626.

71 Jenkins SC, Harris AJ, Lark RM. When unlikely outcomes occur: the role of communication format in maintaining communicator credibility. *J Risk Res* 2018.

72 Gurmankin AD, Baron J, Armstrong K. The effect of numerical statements of risk on trust and comfort with hypothetical physician risk communication. *Med Decis Making* 2004;24:265–271.

73 McDowell M, Rebitschek FG, Gigerenzer G, *et al.* A simple tool for communicating the benefits and harms of health interventions: A guide for creating a Fact Box. *MDM Policy Pract* 2016;1:1–10.

74 McDowell M, Gigerenzer G, Wegwarth O, *et al.* Effect of tabular and icon fact box formats on comprehension of benefits and harms of prostate cancer screening: a randomized trial. *Med Decis Making* 2019;39:41–56.

For peer review only

75 Woloshin S, Schwartz LM, Black WC, *et al.* Cancer Screening campaigns — getting past uninformative persuasion. *N Engl J Med* 2012;367:1677–1679.

76 Schwartz LM, Woloshin S, Floyd J. Fowler J, *et al.* Enthusiasm for Cancer Screening in the United States. *JAMA* 2004;291:71–8.

77 Waller J, Osborne K, Wardle J. Enthusiasm for cancer screening in Great Britain: a general population survey. *Br J Cancer* 2015;112:562–566.

78 Nagler RH. Adverse outcomes associated with media exposure to contradictory nutrition messages. *J Health Commun* 2014;19:24–40.

79 Han PK, Kobrin SC, Klein WM, *et al.* Perceived ambiguity about screening mammography recommendations: association with future mammography uptake and perceptions. *Cancer Epidemiol Prev Biomark* 2007;16:458–466.

80 Cheng C, Dunn M. How well are health information websites displayed on mobile phones? Implications for the readability of health information. *Health Promot J Austr* 2017;28:15–20.

81 Longman T, Turner RM, King M, *et al.* The effects of communicating uncertainty in quantitative health risk estimates. *Patient Educ Couns* 2012;89:252–259.

82 Sladakovic J, Jansen J, Hersch J, *et al.* The differential effects of presenting uncertainty around benefits and harms on treatment decision making. *Patient Educ Couns* 2016;99:974–980.

83 Han PK. Conceptual, methodological, and ethical problems in communicating uncertainty in clinical evidence. *Med Care Res Rev* 2013;70:14S–36S.

84 Ghanouni A, Renzi C, Meisel SF, *et al.* Common methods of measuring ‘informed choice’in screening participation: Challenges and future directions. *Prev Med Rep* 2016;4:601–607.

For peer review only

85 Peters E, Dieckmann N, Dixon A, *et al.* Less is more in presenting quality information to consumers. *Med Care Res Rev* 2007;64:169–190.

86 Waller J, Douglas E, Whitaker KL, *et al.* Women's responses to information about overdiagnosis in the UK breast cancer screening programme: a qualitative study. *BMJ Open* 2013;3:e002703.

87 Hestbech MS, Gyrd-Hansen D, Kragstrup J, *et al.* Effects of numerical information on intention to participate in cervical screening among women offered HPV vaccination: A randomised study. *Scand J Prim Health Care* 2016;34:401–419.

For peer review only

Figure captions

Figure 1. Flowchart representing the results of the website selection after applying exclusion criteria.

Figure 2. Results of analysis of content and quantitative presentation formats for information about (a) cervical screening benefits, (b) risks, (c) possible results, and (d) cervical cancer statistics. A total of 14 websites were analyzed. Items marked with an asterisk were added by the authors to the checklist by Kolthoff et al.[52]. Details concerning instances in which multiple formats were used for a given information item appear in Supplementary materials (Table S3).

For peer review only

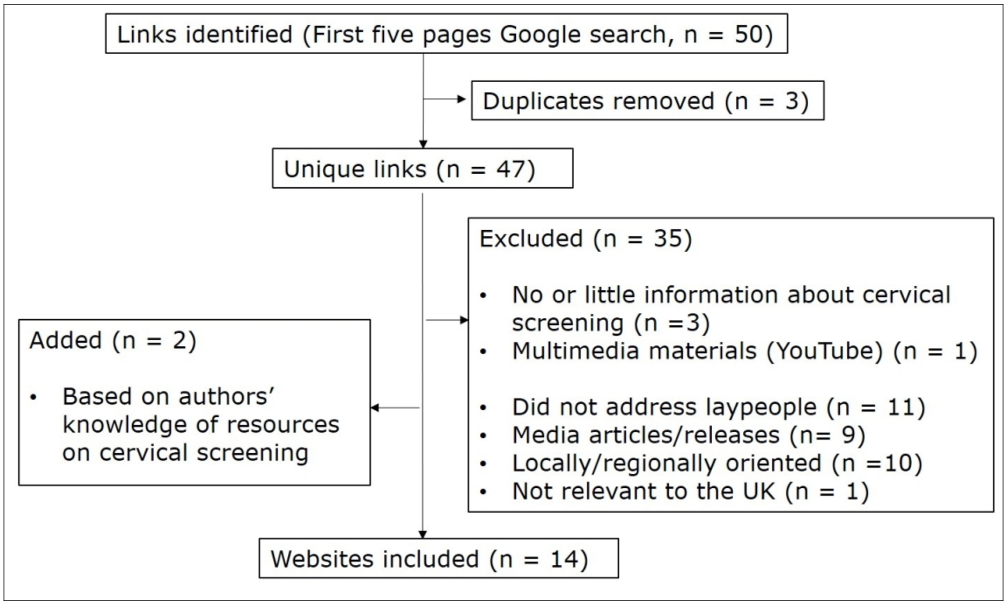

Figure 1. Flowchart representing the results of the website selection after applying exclusion criteria.

241x144mm (150 x 150 DPI)

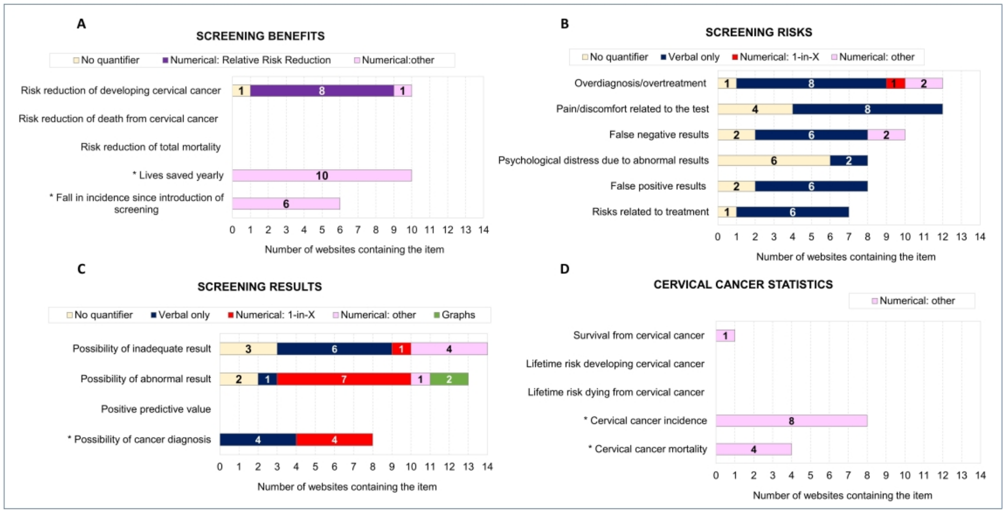

Results of analysis of content and quantitative presentation formats for information about (a) cervical screening benefits, (b) risks, (c) possible results, and (d) cervical cancer statistics. A total of 14 websites were analyzed. Items marked with an asterisk were added by the authors to the checklist by Kolthoff et al.[30]. Details concerning instances in which multiple formats were used for a given information item appear in Supplementary materials (Table S3).

1722x872mm (96 x 96 DPI)

Supplementary materials for BMJ Open

How is cervical screening communicated in UK websites? A cross-sectional analysis of content and quantitative presentation formats

For peer review only

Yasmina Okan,[1 *] Samuel G. Smith,[2] Wändi Bruine de Bruin,[1,3]

[1]Centre for Decision Research, Leeds University Business School, University of Leeds, Leeds, UK

[2] Leeds Institute of Health Sciences, University of Leeds, Leeds, UK

[3]Department of Engineering and Public Policy, Carnegie Mellon University, Pittsburgh, US

* Corresponding author at Centre for Decision Research, Leeds University Business School, University of Leeds, Charles Thackrah Building, Leeds, LS2 9LB, UK; E-mail: y.okan@leeds.ac.uk, Phone: +44 113 343 2622.

Table S1. Websites coded, date of access for analysis, and estimated visits (M=million; K=thousand).

Organization	Link	Date	Total monthly visits [c]	% of UK visitors [c]
British Society for Colposcopy and Cervical Pathology	https://www.bsccp.org.uk/	28/04/2017	- [d]	- [d]
BootsWebMD [a]	https://www.webmd.boots.com/	09/01/2018	- [d]	- [d]
Bupa UK	https:/www.bupa.co.uk	27/04/2017	1.11M	86.15%
Cancer Research UK	https://www.cancerresearchuk.org	27/04/2017	4.46M	47.45%
Jo's Cervical Cancer Trust [a]	https://www.jostrust.org.uk	27/04/2017	205.60K	68.64%
LGBT Foundation [b]	http://lgbt.foundation	27/04/2017	63.18K	66.66%
MacMillan Cancer Support [a]	https://www.macmillan.org.uk	27/04/2017	1.13M	65.03%
Marie Stopes UK [b]	https://www.mariestopes.org.uk	27/04/2017	61.86K	46.77%
NHS Choices (England) [a]	http://www.nhs.uk	20/03/2017	33.53M	71.60%
NHS Inform (Scotland) [a]	https://www.nhsinform.scot	20/03/2017	2.96M	55.43%
Patient Info	https://patient.info	27/04/2017	4.06M	47.05%
Public Health Agency Northern Ireland [a]	http://www.cancerscreening.hscni.net	26/04/2017	402.39K [e]	81.37% [e]
Public Health Wales [a]	http://www.cervicalscreeningwales.wales.nhs.uk	20/04/2017	1.07M [e]	87.68% [e]
Women's Health Concern	https://www.womens-health-concern.org	28/04/2017	116.14K	19.11%

Note: [a] Electronic leaflets and/or factsheets for the general public were available to download via a link this website, and were thus considered part of this resource; [b] Only an electronic leaflet was available and thus the analysis focuses on this resource;[c] Estimated data obtained from SimilarWeb (www.similarweb.com), representing visits in June 2019. Estimates aggregate data across all website subdomains and are computed by extrapolation from a small panel of users, and therefore need to be interpreted with caution; [d] Estimates not available due to insufficient data; [e] Estimates based on the general domain (“hscni.net” and “wales.nhs.uk”)

For peer review only

Table S2. Codes from Kolthoff et al.'s (2016) checklist, with examples of corresponding statements. Codes marked with an asterisk were added by the authors.

**Screening benefits**	
Risk reduction of developing cervical cancer	Regular screening can cut the risk of getting cervical cancer by 75%.
	Cervical screening prevents 8 out of 10 cervical cancers from developing.
Risk reduction of death from cervical cancer	No statements found
Risk reduction of total mortality	No statements found
* Lives saved yearly	Cervical screening saves around 5,000 lives every year in the UK.
	Over 1000 lives are saved every year through regular screening.
* Fall in incidence since introduction of screening	The number of women who develop cervical cancer has halved since the 1980s due to most women regularly having cervical screening.
	Since the screening programme was introduced in the 1980s, the number of cervical cancer cases has decreased by about 7% each year.
**Screening risks**	
Overdiagnosis and overtreatment	The test can pick up minor abnormalities in cervical cells which could clear up by themselves.
	Your screening test may show up mild cell changes in your cervix, which would never have gone on to become cancer or caused you any problems. If this happens, you may receive treatment that you don't really need.
Pain/discomfort related to the test	Cervical screening tests are not painful, although some women find the speculum uncomfortable.
	You might feel some discomfort or pain - try to relax by taking slow, deep breaths as it may hurt more if you are tense.
False negative results	No screening test is 100% accurate. Some tests will be falsely reassuring (so-called false negative results) - where the test is reported as normal but an abnormality is present.
	Regular screening detects up to 80% of cervical abnormalities.
Psychological distress due to abnormal results	A diagnosis of and treatment for cervical abnormalities can cause significant anxiety for many women.
	Many women worry when an abnormality is found.

For peer review only

False positive results	False positive results might wrongly show changes in your cervix.
	Very occasionally, screening can show you have abnormal cells when in fact there's no problem. This is called a false positive result.
Risks related to treatment	For a few women, the treatments may cause problems such as bleeding afterwards or a small increase in future pregnancies of having the baby early.
	Very rarely, the cervix can become tightly closed after treatment. This known as stenosis. It can make it harder for the sperm to enter the womb and so can affect your chances of becoming pregnant naturally.
**Screening results**	
Possibility of inadequate result	Sometimes test results are declared 'unsatisfactory' because the slide was poorly prepared or difficult to read. In such cases, the test needs to be repeated and the patient will be called back.
	An inadequate result does not mean your smear was abnormal but that it was unreadable. This means that there were not enough cells in the sample and happens in about 3 out of every 100 tests taken.
Possibility of abnormal result	Most abnormal results from screening tests show only very minor changes.
	About 1 in 20 women will have a smear test result which is abnormal.
Positive predictive value	No statements found
* Possibility of cancer diagnosis	A cervical screening test can very occasionally find early cervical cancer. Most women with an abnormal test result have early cell changes and not cancer.
	It is extremely rare for cancer to be diagnosed from a cervical screening test. Less than one in 1,000 women's test results show invasive cancer.
**Cervical cancer statistics**	
Survival from cervical cancer	Overall, almost 2 out of 3 women (63%) survive ten years or more, and more than 8 out of 10 women (83%) will live at least one year.
Lifetime risk of developing cervical cancer	No statements found
Lifetime risk of dying from cervical cancer	No statements found
* Cervical cancer incidence	Around 3,000 women are diagnosed with cancer of the cervix every year.
	Each year in Northern Ireland, about 103 women are diagnosed with cervical cancer.
* Cervical cancer mortality	Sadly around 900 women die of cervical cancer each year in England.
	In Northern Ireland, 20–30 women die each year from cervical cancer.

For peer review only

Table S3a. Information items about cervical screening benefits, risks, results, and cervical cancer statistics (Part 1)

	BSCCP	Boots WebMD	Bupa UK	Cancer Research UK	Jo's Cervical Cancer Trust	LGBT Foundation	MacMillan Cancer Support
**Screening benefits**							
Risk reduction of developing cervical cancer	-	-	-	RR	RR	RR	0
Risk reduction of death from cervical cancer	-	-	-	-	-	-	-
Risk reduction of total mortality	-	-	-	-	-	-	-
* Lives saved yearly	-	-	N	N	N	-	N
* Fall in incidence since the introduction of screening [a]	N	-	-	N	-	-	N
**Screening risks**							
Overdiagnosis and overtreatment	V	-	V	V	V, N	V	V
Pain/discomfort related to the test	-	0	0	0	V	V	V
False negative results	-	-	0	0	V, N	-	V
Psychological distress due to abnormal results	0	-	0	0	V	V	0
False positive results	V	-	V	V	-	-	V
Risks related to treatment	V	-	-	V	V	-	V
**Screening results**							
Possibility of inadequate result	0	V	V, N	V	V	0	V
Possibility of abnormal result	1X, N	0	1X	1X, N	V, 1X	V, 1X	V
Positive predictive value	-	-	-	-	-	-	-
* Possibility of cancer diagnosis	V, 1X, N	-	-	V	V, 1X	V	V
**Cervical cancer statistics**							
Survival from cervical cancer	-	N	-	-	-	-	-
Lifetime risk of developing cervical cancer	-	-	-	-	-	-	-
Lifetime risk of dying from cervical cancer	-	-	-	-	-	-	-
* Cervical cancer incidence	-	N	N	-	N	-	-
* Cervical cancer mortality	-	-	-	-	N	N	-
**Type of appeals**							
Participation	x	x	x	x	x	x	x
Informed decision making	-	-	x	-	x	-	-

Note: - = item not present; 0 = not quantified; V = verbal quantifier; RR = numerical relative risk reduction; 1X = numerical 1-in-X; N = numerical (other); G = graph; x = item present (coding format not applicable); * codes added by the authors; [a] includes estimates of incidence at different time points, as well as any mention to the magnitude of the fall in incidence (e.g., rates have halved).

For peer review only

Table S3b. Information items about cervical screening benefits, risks, results, and cervical cancer statistics (Part 2)

	Marie Stopes UK	NHS Choices (England)	NHS Inform (Scotland)	Patient Info	PHA Northern Ireland	Public Health Wales	Women's Health Concern
**Screening benefits**							
Risk reduction of developing cervical cancer	RR	N	RR	RR	RR	RR	-
Risk reduction of death from cervical cancer	-	-	-	-	-	-	-
Risk reduction of total mortality	-	-	-	-	-	-	-
* Lives saved yearly	N	N	N	N	-	N	N
* Fall in incidence since the introduction of screening [a]	-	N	-	N	-	N	-
**Screening risks**							
Overdiagnosis and overtreatment	0	V	V	V, N	-	V, 1X	V
Pain/discomfort related to the test	V	V	-	V	V	V	0
False negative results	-	V	V	V	V	V	V, N
Psychological distress due to abnormal results	-	0	-	0	-	-	-
False positive results	-	V	0	0	-	V	-
Risks related to treatment	-	V	-	-	0	V	-
**Screening results**							
Possibility of inadequate result	0	V, N	V	N	V, N	1X, N	V
Possibility of abnormal result	0	V, 1X, N, G	V, 1X, G	V, N	V, 1X, N	V, 1X, N	-
Positive predictive value	-	-	-	-	-	-	-
* Possibility of cancer diagnosis	-	V, 1X	-	V, 1X	-	V	-
**Cervical cancer statistics**							
Survival from cervical cancer	-	-	-	-	-	-	-
Lifetime risk of developing cervical cancer	-	-	-	-	-	-	-
Lifetime risk of dying from cervical cancer	-	-	-	-	-	-	-
* Cervical cancer incidence	N	N	-	N	N	N	-
* Cervical cancer mortality	-	-	-	-	N	N	-
**Type of appeals**							
Participation	-	-	x	x	x	x	x
Informed decision making	-	x	x	-	-	x	x

Note: - = item not present; 0 = not quantified; V = verbal quantifier; RR = numerical relative risk reduction; 1X = numerical 1-in-X; N = numerical (other); G = graph; x = item present (coding format not applicable); * codes added by the authors; [a] includes estimates of incidence at different time points, as well as any mention to the magnitude of the fall in incidence (e.g., rates have halved).

For peer review only

BMJ Open

How is cervical cancer screening information communicated in UK websites? A cross-sectional analysis of content and quantitative presentation formats

Journal:	*BMJ Open*
Manuscript ID	bmjopen-2019-029551.R2
Article Type:	Original research
Date Submitted by the Author:	03-Sep-2019
Complete List of Authors:	Okan, Yasmina; University of Leeds, Centre for Decision Research, Leeds University Business School<br>Smith, Samuel; University of Leeds, Leeds Institute of Health Sciences<br>Bruine de Bruin, Wändi; University of Leeds, Centre for Decision Research, Leeds University Business School; Carnegie Mellon University, Department of Engineering and Public Policy
**Primary Subject Heading**:	Communication
Secondary Subject Heading:	Public health
Keywords:	cervical cancer screening, informed decision making, risk communication, risk perception, web analysis

SCHOLARONE™
Manuscripts

Running Head: CERVICAL CANCER SCREENING WEBSITES

Word count: 3,939

How is cervical cancer screening information communicated in UK websites? A cross-sectional analysis of content and quantitative presentation formats

Yasmina Okan,[1,*] Samuel G. Smith,[2] Wändi Bruine de Bruin,[1,3]

[1]Centre for Decision Research, Leeds University Business School, University of Leeds, Leeds, UK

[2] Leeds Institute of Health Sciences, University of Leeds, Leeds, UK

[3]Department of Engineering and Public Policy, Carnegie Mellon University, Pittsburgh, US

* Corresponding author at Centre for Decision Research, Leeds University Business School, University of Leeds, Charles Thackrah Building, Leeds, LS2 9LB, UK; E-mail: y.okan@leeds.ac.uk, Phone: +44 113 343 2622.

For peer review only

Abstract

Objectives: To investigate whether UK websites about cervical cancer screening targeted to the public include (1) information about benefits and risks of screening, possible screening results, and cervical cancer statistics, (2) quantitative presentation formats recommended in the risk communication literature, and (3) appeals for participation and/or informed decision making.

Design: Cross-sectional analysis of websites using a comprehensive checklist of information items on screening benefits, risks, possible results, and cervical cancer statistics.

Outcome measures: We recorded the number of websites that contained each of the information items, and the presentation format used for probabilistic information (no quantification provided, verbal quantifiers only, different types of numerical formats, and/or graphs). We also recorded the number of websites containing appeals for participation and/or informed decision making.

Setting: Websites were identified through the most common Google search terms used in the UK to find information on cervical screening, according to GoogleTrends and a commercial internet-monitoring programme. Two additional websites were identified by the authors as relevant.

Results: After applying exclusion criteria, 14 websites were evaluated, including websites of public and private health service providers, charities, a medical society, and a pharmacy. The websites mentioned different benefits, risks of screening, and possible results. However, specific content varied between websites. Probabilistic information was often presented using non-recommended formats, including relative risk reductions to express screening benefits, and verbal quantifiers without numbers

For peer review only

to express risks. Appeals for participation were present in most websites, with almost half also mentioning informed decision making.

Conclusions: UK websites about cervical cancer screening were generally balanced. However, benefits and risks were presented using different formats, potentially hindering comparisons. Additionally, recommendations from the literature to facilitate understanding of quantitative information and facilitate informed decisions were often not followed. Designing websites that adhere to existing recommendations may support informed screening uptake.

Keywords: cervical cancer screening; informed decision making; risk communication; risk perception; web analysis

For peer review only

Article summary

Strengths and limitations of this study

- We analyzed the content of UK websites about cervical cancer screening using an established checklist of information items, and identified additional information that was mentioned commonly.
- We systematically examined whether cancer screening websites present probabilistic information in formats recommended in the risk communication literature.
- We identified websites by applying the most common Google search terms used in the UK to find information on cervical screening, and examined the majority of links that users will realistically access.
- All the information items we assessed may not be relevant for screening decisions, whereas we may have omitted some information that can be relevant (e.g., about the human papillomavirus).
- We did not examine whether websites mentioned the uncertainty associated with estimates of benefits and risks, although such information can be important for informed decision making.

For peer review only

INTRODUCTION

Cervical cancer is highly preventable. It is caused in most cases by an infection with the human papillomavirus (HPV), which may lead to abnormal changes in cervical cells[1,2]. Such abnormalities can be detected through screening and treated before they become cancerous[3]. Indeed, cervical screening significantly reduces both cervical cancer incidence and cancer-specific mortality[3–7]. In the UK, age-standardized incidence was 11.8 in 100,000 in 2014, and age-standardized mortality was 3.3 in 100,000[8]. In England alone, estimates suggest that there would be 1,827 additional cervical cancer deaths per year without screening[7].

However, cervical screening is associated with potential risks, including the detection of indolent abnormal cells that would have cleared up on their own (i.e., overdiagnosis), potentially leading to unnecessary treatment[9,10]. A recent meta-analysis indicates that about half of untreated moderately abnormal cells regress within two years[9], suggesting that overtreatment is relatively common. The removal of large amounts of tissue during treatment can also increase the risk of preterm birth in future pregnancies[11–13]. However, experts and policy makers generally agree that benefits of cervical screening outweigh potential risks[5,14,15]. In the UK, the National Health Service (NHS) offers screening to all women aged 25-64. It has been emphasized that screening invitees need information about both benefits and risks to make informed decisions about participation[14,16,17].

Organized screening programs often use written outreach materials. In the UK, eligible women receive an invitation letter with an information leaflet that mentions websites about cervical screening. Hence it is important to examine whether widely accessed websites effectively support decisions about screening participation. Both European and UK guidelines have emphasized that cancer screening

For peer review only

communications should be comprehensive in content, and should provide balanced discussions of benefits and risks to screening[17–20]. Such guidelines have also highlighted that screening communications should be comprehensible, and avoid quantitative presentations that are hard to understand[17–19]. Communications that are not well understood can cause undue concern, reduce recipients' self-efficacy beliefs about their capacity to participate in screening, and undermine informed decision making[21,22].

Quantitative information in screening communications, however, can be challenging even for educated audiences[21,23]. Such information can be presented using a range of formats, including verbal quantifiers (e.g., "low" or "moderate" risk), various numerical formats, and graphical displays (e.g., icon arrays). Research in risk communication has shown that quantitative presentation formats vary considerably in their effectiveness to promote accurate understanding. Some formats are known to be relatively ineffective, while others still lack conclusive evidence[24]. Table 1 provides an overview of quantitative formats, associated evidence-based recommendations and supporting references. Key recommendations relevant to screening communications include avoiding the use of verbal quantifiers without accompanying numbers; numerical "1-in-X" formats; and presentations of risk reductions in relative terms. Another recommendation is to add simple graphical displays to numerical information.

For peer review only - http://bmjopen.bmj.com/site/about/guidelines.xhtml

Table 1. Evidence-based recommendations from the risk communication literature to promote understanding of probabilistic information

Recommendation	Rationale	Key references (first author)
Avoid the use of verbal quantifiers without numbers (e.g., women who have abnormal cells removed are *slightly more likely* to have their baby early").	Interpretations of verbal quantifiers vary across individuals and often differ from interpretations intended by communicators. Verbal quantifiers can lead to overestimations of risks.	Budescu [25]; Knapp [26,27]; Peters [28]; Visschers et al. [29]; Young [30]; Zipkin [31]
Avoid numerical "1-in-X" formats to present the chance of an outcome (e.g., 1 in 12 women will have an abnormal test result).	People tend to perceive the same probabilities as higher and more worrying when presented in a "1-in-X" format, as compared to numerically equivalent "N-in-X*N" ratios (e.g., 10 in 120 women will have an abnormal test result) or percentages (e.g., 5% women will have an abnormal test result).	Pighin [32,33]; Sirota [34–36]; Trevena [37]; Zikmund-Fisher [38]
Avoid presenting estimates of risk reduction in relative terms (e.g. screening cuts the risk of getting cervical cancer by 75%).	Relative risk differences can obscure the true magnitude of benefit or harm, as compared to absolute risk differences (e.g., screening reduces the risk of getting cervical cancer from 20 in 1,000 to 5 in 1,000).	Akl [39]; Covey [40]; Fagerlin [22], Gigerenzer [21,41]; Trevena [37] Zipkin [31]

For peer review only

Add simple graphical displays of numerical information (e.g., icon arrays, where icons of different colors represent those affected and not affected by the risk).	Well-designed, simple graphs help to overcome difficulties in understanding numerical information and are often perceived as more appealing and easier to understand.	Galesic [42]; Garcia-Retamero [43]; Gigerenzer [41]; Okan [44]; Paling [45]; Schapira [46]

For peer review only

Existing guidelines also state that screening communications should not persuade people to attend or present screening as necessary or important, without acknowledging that not participating is a reasonable choice[17–20]. Recent guidance from the UK National Screening Committee[17] notes that "information should make it clear that it is a personal choice to accept or decline screening and both choices will be fully supported" (p. 6). This approach to screening communications seeks to respect personal autonomy and ensure that decisions are in line with invitees' personal values and circumstances – especially since healthy individuals can be adversely affected by screening[47,48]. Besides undermining the principle of autonomy, persuasive messages may have unintended negative effects, such as eliciting guilt and anxiety among invitees who decline the offer, anger among those who participate and are harmed as a result, and potential mistrust in communicators over time[20,48,49].

We investigated whether UK websites about cervical screening adhere to existing guidelines and policy recommendations concerning information content and appeals for informed decision making (vs. for participation), as well as to recommendations from the risk communication literature concerning quantitative presentation formats. Previous website analyses have primarily focused on breast cancer screening, and have generally assessed only website content[47,50] or a specific aspect of presentation format (e.g., consistency in the presentation of statistics on overdiagnosis[51]).

We used an established checklist of information items about cervical screening[52], building on earlier evaluations of breast cancer screening communications[47,53,54]. We assessed quantitative presentation formats

For peer review only

considering the recommendations listed in Table 1. Additionally, we examined the type of appeals included in websites. In sum, our research questions were:

1. Do UK websites about cervical screening contain a) key information about screening benefits, risks, possible results, and b) cervical cancer statistics?
2. Do UK websites about cervical screening present probabilistic information using recommended formats?
3. Do UK websites about cervical screening contain appeals for participation and/or informed decision making?

METHODS

Search strategy

We used GoogleTrends to identify the most common Google search terms used in the UK to find information on cervical screening. We identified search terms related to "cervical screening" in the 12 months prior to March 2017. We used "cervical screening" based on the screening invitations from the UK's National Health Service. Related search terms included "cervical cancer screening," "cervical smear," and "smear test." To determine which of these terms was more common, we used Wordtracker (https://app.wordtracker.com/), a commercial program that estimates the relative frequency of Google search terms in a given period of time (see[55]for a similar procedure). The two most commonly used terms in the UK in the year before March 2017 were "smear test" and "cervical screening."

Website selection

On March 9th, 2017, we performed the Google search '"smear test" OR "cervical screening"' using the private Firefox browsing mode to prevent previously visited pages and cookies from influencing search results. Following Ghanouni et al.[51], we examined the first five pages of results using the default of 10 results per page,

For peer review only - http://bmjopen.bmj.com/site/about/guidelines.xhtml

resulting in 50 links. This includes the vast majority of websites that users will access (see also[56]). The full list of links is available at the Open Science Framework (https://doi.org/10.17605/OSF.IO/73GFN).

We excluded links that (1) targeted healthcare professionals and academics rather than laypeople (e.g., technical reports, research articles), (2) reflected online media articles or press releases, (3) presented international information not applicable to the UK (e.g., Wikipedia's overview of screening recommendations in different countries), and (4) were locally or regionally-oriented. The latter links typically focused on basic practical aspects (e.g., who is eligible for screening and logistics of making appointments for specific GP surgeries), and often provided links for further information to national websites included in our analyses. We also excluded links that (5) were duplicate, (6) contained no or little information about cervical screening (<150 words), (7) had no written materials (e.g., YouTube video). Additionally, we included two websites identified through our knowledge of resources on cervical screening (Patient and Women's Health Concern). Although these websites did not appear in the Google search, they represent trusted UK resources that some women may access directly to learn about cervical screening. Figure 1 summarizes the website selection process.

For the websites identified through Google, we coded the link listed in the search results. For the two additional websites, we coded the main link providing information about cervical screening. In all cases, we also coded sections on cervical screening within each website that could be directly accessed from the initial link, as well as other written materials directly accessible, including electronic leaflets, fact sheets, and slideshows, which were all considered as part of the same resource in analyses. Websites were accessed between 20th March 2017 and 28th April 2017, except one

For peer review only

website (BootsWebMD), which was accessed at a later date due to an oversight in the initial website selection. The date of accessing each website, corresponding links, and estimated number of visits appears in Supplementary materials (Table S1). Copies of PDF files reflecting the content of all websites at the time of access are available at the Open Science Framework.

Coding of websites

To code website content, we adapted a checklist of information items that was developed to analyze invitations for cervical screening[52]. Following European and UK guidelines[18,19], the checklist included items about screening benefits, risks, and possible results (e.g., the possibility of an abnormal or an inadequate result). Following Kolthoff et al.[52] the item on overdiagnosis/overtreatment included any reference to screening possibly detecting abnormal cells that may clear up on their own and/or leading to unnecessary treatment, regardless of whether the treatment type was mentioned (see also[57,58]). The checklist also included items about cervical cancer statistics (e.g., lifetime risk of developing cervical cancer), which may be relevant for screening decisions[47,53,54]. After initial inspection of the websites, we added five items to the checklist to reflect commonly presented information that may also influence screening decisions (Figure 2). Examples of all information items appear in Supplementary materials (Table S2).

To assess quantitative presentation formats, we coded whether probabilistic information was presented using: no quantifiers, verbal quantifiers only, numerical quantifiers (1-in-X vs. other numerical format), and/or graphs. We also coded whether any information about risk reduction was presented in relative terms. Multiple formats (e.g., graphs accompanied by numbers) were present in some communication materials (Table S3, Supplementary materials).

For peer review only

Finally, we coded appeals for informed decision making (e.g., “deciding whether to have screening or not is your choice”) and appeals for participation. Following Kotlhoff et al.[52], the latter included direct encouragement for participation (e.g., “a smear test—have it”) and statements presenting screening as necessary (e.g., “it’s essential for women to have regular cervical screening tests”) or important (e.g., “it is important to go for screening every time you are invited”).

The first author (YO) read through all selected materials and applied the coding scheme. Three websites (>20%) were coded by another author (SS). Cohen’s kappa was moderate to high on all codes except ‘article tone’ (.18). Discrepancies for this code were caused by appeals for participation that were not prominent. These discrepancies were resolved through discussion, resulting in consensus. The average Cohen’s kappa was .86 (range: .65 to 1).

Patient and public involvement

Patients and/or public were not involved in the design or conduct of the study, which did not involve human participants.

RESULTS

Website selection

As described above, we identified 50 links through our Google search. After exclusions, 12 websites remained, including 4 websites from public health service providers in the UK’s four countries (NHS Choices England, NHS Inform Scotland, Public Health Agency Northern Ireland, Public Health Wales), one private health service provider (Bupa UK), three cancer charities (Cancer Research UK, Jo’s Cervical Cancer Trust, MacMillan Cancer Support), two additional charities (LGBT Foundation, Marie Stopes UK), one medical society (British Society for Colposcopy and Cervical Pathology), and one pharmacy (Boots WebMD). All websites from

For peer review only

public health service providers included links to official invitation leaflets, which were coded with the corresponding website, as described above. As noted earlier, two additional websites (Patient and Women's Health Concern) were identified by the authors. Below, we present summary statistics across websites. Results for individual websites appear in Supplementary materials (Table S3).

<Figure 1>

Do UK websites about cervical screening contain a) key information about screening benefits, risks, possible results, and b) cervical cancer statistics?

We report on four main findings regarding website content. First, screening benefits were mentioned frequently, although the focus was primarily on risk reduction of developing cervical cancer (Figure 2a). Estimates of risk reduction typically varied between 70% and 80%. Based on Sasieni et al.[59], two websites provided an estimated range of 60-80%, and another provided an estimate of 90% for the reduction in the risk of advanced cancer specifically. No websites explicitly mentioned risk reductions of death from cervical cancer or total mortality. Instead, benefits were often expressed in terms of the number of lives saved yearly (e.g., "cervical screening saves 5,000 lives a year in the UK"; n=10 websites), or in some cases referring to the fall in cervical cancer cases since the national screening programme was initiated (e.g., "since the screening programme was introduced in the 1980s, the number of cervical cancer cases has decreased by about 7% each year"; n=6).

Second, screening risks were also mentioned relatively frequently (Figure 2b), but the specific risks and their descriptions varied across websites. The most commonly mentioned risks were overdiagnosis and overtreatment (n=12), pain/discomfort related to the test (n=12), and the possibility of false negatives

For peer review only

(n=10). Descriptions of overdiagnosis, however, generally focused on cell changes often clearing up and not progressing into cancer (e.g., “in most cases, the abnormal cells will disappear on their own”). The risk of unnecessary additional tests or treatment was only mentioned explicitly in seven cases, often in connection to justifications for current screening age ranges (e.g., “changes in a young woman’s cervix are quite normal. In this situation, screening may lead to unnecessary treatment”). Most websites described the test as uncomfortable but not painful (e.g., “having the speculum put in may be a little uncomfortable, but it shouldn’t hurt”), though one website did state that the test was potentially painful. Risks related to treatment were only mentioned in half of the websites, all of which referred to the possibility of premature birth. Two of them also noted the risk of stenosis (i.e., the cervix becoming tightly closed after treatment).

Third, possible screening results were mentioned in most websites (Figure 2c). Estimates of the likelihood of inadequate results varied between 1.7 and 3% and estimates of the likelihood of abnormal results ranged between 5 and 10%. Additionally, the possibility of cancer diagnosis was mentioned in over half of the websites (n=8) and was often reported as occurring in less than 1 in 1,000 cases.

Finally, cervical cancer statistics such as lifetime risk of developing or dying from cervical cancer were not discussed, although some websites did provide details on cervical cancer incidence and mortality (Figure 2d). Specific estimates of incidence varied across websites, and included yearly, daily, national, and regional estimates (e.g., “each year in Northern Ireland, about 103 women are diagnosed with cervical cancer”).

<Figures 2a, 2b, 2c, and 2d>

For peer review only

Do UK websites about cervical screening present probabilistic information using recommended formats?

Information was often not presented in recommended formats. First, the recommendation to avoid the use of verbal quantifiers without numbers[31] was often not followed for information about risks (Figure 2b). Risks related to treatment were quantified only verbally, generally using varying verbal quantifiers. For example, one website stated that "women are *slightly more likely* to have their baby 1 to 2 months early," and another stated that "women may have a *higher risk* of preterm delivery." Other risks were also either only quantified verbally or not quantified at all, with the exception of overdiagnosis/overtreatment (n=3) and false negative results (n=2).

Second, the recommendation to avoid "1-in-X" numerical formats[38] was often violated for information about screening results. This was the most popular format for conveying the likelihood of abnormal results (Figure 2c). Third, the recommendation to avoid presentations of relative risk reduction[21] was violated in most cases in which an estimate of risk reduction was provided (Figure 2a). Finally, the recommendation to add simple graphical displays[43] was often not met. Only two websites contained graphs, each depicting the likelihood of abnormal results.

Do UK websites about cervical screening contain appeals for participation and/or informed decision making?

Appeals for participation were present in most websites (n=12). Specifically, appeals for participation without mention of informed decision making occurred in half of the websites (n=7). Several websites combined appeals for participation and informed decision making (n=5) (e.g., "don't ignore your smear test, it could save your life" and "taking part in cervical screening is your choice"). One website (NHS

For peer review only

Choices England) referred to informed decision making only ("deciding whether or not to have a cervical screening test is your choice"). The websites of the three remaining public health service providers included either both appeals for participation and informed decision making (NHS Inform Scotland and Public Health Wales) or only appeals for participation (Public Health Agency Northern Ireland).

DISCUSSION

We investigated whether UK websites about cervical screening adhered to existing recommendations about content, quantitative presentation formats, and type of appeals. We found that websites often followed recommendations from European and UK guidelines to include information about both benefits and risks of screening[17–19]. However, specific content varied across websites. For example, only half of the websites explicitly referred to risks related to the treatment of abnormal cells. Additionally, probabilistic information was often presented in formats not recommended in the risk communication literature, including relative risk reductions to express screening benefits, and verbal quantifiers without numbers to express risks. Moreover, several websites encouraged screening participation without referring to informed decision making, contrasting with current UK policy[14,60].

Our finding that websites often included information about both benefits and risks of cervical screening contrasts with previous reports that breast screening websites lacked information about risks such as overdiagnosis[47,50]. Recent emphasis in the UK on facilitating informed screening decisions[14,60] may have led to more comprehensive and balanced websites in recent years (see also[51]). The presence of information about both screening benefits and risks is aligned with women's preferences, who often want to receive information on both aspects before their

For peer review only

test[61]. However, we also found that websites generally presented benefits and risks using different formats (numbers vs. verbal quantifiers, respectively), potentially hindering benefit-risk comparisons. Additionally, our finding that specific content varied across websites suggests that women accessing different resources may come to different conclusions.

Our results also suggest that existing descriptions of benefits and risks may not always support understanding. For example, the concepts of overdiagnosis and overtreatment are often unfamiliar and counterintuitive to people[62–64], highlighting the need to provide explanations that are accessible and transparent. Many websites did explain that cell changes often clear up on their own. This may help women to understand that cervical screening can lead to overdiagnosis of indolent abnormalities (potentially causing unnecessary anxiety or worries[58]), but not necessarily that it may result in unnecessary treatment. Moreover, the websites that explicitly mentioned overtreatment often did so in relation to women below the recommended screening age. Hence, those within the recommended age may incorrectly infer that such risk does not apply to them. We also found that many websites emphasized that screening “saves lives.” It has been argued that such claims are misleading, and that communications should report reductions in cancer-specific mortality, overall mortality, as well as overall cancer deaths[65]. Although there is evidence that cervical screening reduces cancer-specific mortality[3–7], determining its impact on overall mortality requires large trials with sufficient power to detect differences[66].

Our finding that probabilistic information was often presented in non-recommended formats is concerning as this may cause misperceptions, even among educated audiences. The use of relative risk reductions to express screening

For peer review only - http://bmjopen.bmj.com/site/about/guidelines.xhtml

benefits can hinder understanding and increase risk perceptions, relative to presentations of absolute risk reduction[31,37]. Although information about baseline risk (i.e., the absolute risk without screening) could reduce the biasing effect of relative risks[67] (but see [68]), such beneficial impact may be limited to baseline risks presented in frequency formats (vs. probabilities)[69,70] and to more (vs. less) numerate recipients[70]. Moreover, we did not find such information in any of the websites. It has been argued, however, that presentations of relative risks may be considered for low-probability risks with high impact (e.g., an earthquake), to prevent people from neglecting such risks altogether[24,29].

Additionally, our results suggest that women may overestimate the likelihood of abnormal screening results due to the use of "1-in-X" formats (e.g., "1 in 12 women will have an abnormal test result")[36]. Similarly, screening risks expressed using verbal quantifiers without numbers may be overestimated[26–28,30]. The absence of numerical information can also lead people to perceive communications as less credible and trustworthy[29,71,72]. Although some risks are hard to quantify (e.g., psychological distress), estimates are available for risks such as preterm birth or overtreatment, from observational studies and meta-analyses[9,11,13]. The best available evidence could be presented in transparent fact boxes—a tabular format that facilitates comparisons of outcomes in groups of screened vs. unscreened individuals[73]. Fact boxes can also include simple graphs such as icon arrays to allow visual comparison of quantities[74], which could be especially beneficial for people with low numeracy. Yet, graphs were seldom used in cervical screening websites.

Finally, our finding that some websites encouraged screening without mentioning informed decision making contrasts with current UK policy, which emphasizes that

For peer review only

communications can recommend screening, but should acknowledge that not accepting the offer is a reasonable choice[14,20,60]. Although there is largely agreement that benefits of cervical screening outweigh potential risks[5,14,15], persuasive messages raise ethical concerns to the extent that harms are possible[18,48,75]. Persuasive messages may also contribute to widespread enthusiasm for cancer screening (e.g.,[76,77]), discouraging people from reasoning about their screening choices[48]. Moreover, while guidelines for screening communications tend to focus on health service providers[17–19], conflicting recommendations from different sources might create public confusion, negative beliefs about recommendations and scientific research, and potentially reduce screening intentions[78,79].

Strengths of our analyses include their comprehensiveness and systematic examination of website content using an established checklist of information items[52], and recommendations from the literature for quantitative presentation formats. Additionally, our analyses included most of the websites that UK citizens may realistically access, as our strategy for website selection was based on the top results identified by the most commonly used Google search terms. Our approach could be used by researchers and practitioners to evaluate the content and format of websites about related topics (e.g., other types of screening) in different countries. This would allow comparisons of websites for countries that have organized vs. opportunistic screening. For example, website content may be more heterogeneous in countries such as the United States, which do not have organized screening. It would also be interesting to compare different platforms (e.g., desktop websites vs. mobile tailored versions), and to examine whether different design features (e.g.,

For peer review only

information position[80]) affect information readability, saliency, or users' information seeking behavior .

Limitations of our study include that our analyses may not have covered all information that is potentially relevant for screening decisions. For example, we did not assess whether websites discussed the human papillomavirus (HPV), or the uncertainty associated with estimated benefits and risks. Communicating uncertainty can be challenging[81–83], but is important for informed decision making[16,17,60]. Conversely, it is possible that not all codes assessed are essential for informed screening decisions. We also did not directly examine whether users can make informed screening decisions based on the different websites. It is likely, however, that following guidelines and recommendations from the literature will facilitate better understanding and more informed decisions. Explicit appeals to informed decision making may also encourage users to evaluate the content, but of course do not guarantee that informed decisions will occur.

Future work should aim to identify essential information items for informed decisions about screening, considering both experts' and women's views. As noted by Ghanouni et al.[84], guidelines often do not provide detailed advice on *which* specific screening benefits, risks, or results should be discussed in communications. Moreover, experts may not fully agree on the relative importance of different information items, and women may have different information needs and preferences, including about whether and when to receive information about further tests. Providing too much unfamiliar information initially may also overwhelm screening invitees, and potentially distract them from key information necessary for decision making[22,85]. More research is also needed to identify how to best convey the individual significance of the population-level estimates included in screening

For peer review only

communications, which may be hard for people to understand[86]. Finally, future studies should also examine how screening decisions (e.g., intentions to participate[87]) are affected by information about specific benefits and/or risks presented using different formats, building on the risk communication literature (e.g., [88]). Such work would provide valuable insights to inform the design of evidence-based public communications about screening.

For peer review only

Acknowledgements

We are grateful to Mirta Galesic and Erika Waters for their valuable comments and suggestions. We would also like to thank the reviewers (Jo Waller, Felix G. Rebitschek, and Seok Won Jin) for their constructive feedback.

Funding

This work was supported by a Population Research Fellowship awarded by Cancer Research UK to Yasmina Okan (reference C57775/A22182). Samuel G. Smith is supported by Yorkshire Cancer Research. Wändi Bruine de Bruin was partially supported by a grant from the Swedish Riksbanken Jubilieumsfond programme on Science and Proven Experience. The funding agencies had no involvement in designing the study, data collection, analysis and interpretation, writing the report, or the decision to submit the article for publication.

Competing interests

SS is an academic consultant for Luto, who are not involved in any of the websites reviewed in this manuscript.

Author contributions

YO conceived the research, acquired and analysed the data, and drafted the initial version of the manuscript. SS assisted with data analyses and interpretation. WBB contributed to data interpretation. All authors contributed to study design, revised the manuscript critically and approved the final version.

Data sharing statement

The full list of links initially identified and copies of PDF files reflecting the content of all analyzed websites at the time of access are available at the Open Science Framework (https://doi.org/10.17605/OSF.IO/73GFN).

For peer review only

References

1 Crosbie EJ, Einstein MH, Franceschi S, *et al.* Human papillomavirus and cervical cancer. *The Lancet* 2013;382:889–99.

2 Muñoz N, Bosch FX, de Sanjosé S, *et al.* Epidemiologic classification of human papillomavirus types associated with cervical cancer. *N Engl J Med* 2003;348:518–27.

3 Peirson L, Fitzpatrick-Lewis D, Ciliska D, *et al.* Screening for cervical cancer: A systematic review and meta-analysis. *Syst Rev* 2013;2:35.

4 Nygård M. Screening for cervical cancer: when theory meets reality. *BMC Cancer* 2011;11:240.

5 Esserman LJ, Thompson IM, Reid B. Overdiagnosis and overtreatment in cancer: an opportunity for improvement. *J Am Med Assoc* 2013;310:797–798.

6 Peto J, Gilham C, Fletcher O, *et al.* The cervical cancer epidemic that screening has prevented in the UK. *Lancet* 2004;364:249–256.

7 Landy R, Pesola F, Castañón A, *et al.* Impact of cervical screening on cervical cancer mortality: Estimation using stage-specific results from a nested case–control study. *Br J Cancer* 2016;115:1140–1146.

8 Smittenaar CR, Petersen KA, Stewart K, *et al.* Cancer incidence and mortality projections in the UK until 2035. *Br J Cancer* 2016;115:1147–1155.

9 Tainio K, Athanasiou A, Tikkinen KA, *et al.* Clinical course of untreated cervical intraepithelial neoplasia grade 2 under active surveillance: Systematic review and meta-analysis. *Br Med J* 2018;360:k499.

10 McCredie MR, Sharples KJ, Paul C, *et al.* Natural history of cervical neoplasia and risk of invasive cancer in women with cervical intraepithelial neoplasia 3: A retrospective cohort study. *Lancet Oncol* 2008;9:425–434.

For peer review only

11 Castanon A, Landy R, Brocklehurst P, *et al.* Risk of preterm delivery with increasing depth of excision for cervical intraepithelial neoplasia in England: Nested case-control study. *Br Med J* 2014;349:g6223.

12 Sasieni P, Castanon A, Landy R, *et al.* Risk of preterm birth following surgical treatment for cervical disease: executive summary of a recent symposium. *BJOG Int J Obstet Gynaecol* 2016;123:1426–1429.

13 Kyrgiou M, Athanasiou A, Kalliala IEJ, *et al.* Obstetric outcomes after conservative treatment for cervical intraepithelial lesions and early invasive disease. *Cochrane Database Syst Rev* 2017;11:CD012847.

14 Forbes LJ, Ramirez AJ. Communicating the benefits and harms of cancer screening. *Curr Oncol Rep* 2014;16:382.

15 Moyer VA. Screening for cervical cancer: US Preventive Services Task Force recommendation statement. *Ann Intern Med* 2012;156:880–891.

16 Hersch JK, Nickel BL, Ghanouni A, *et al.* Improving communication about cancer screening: Moving towards informed decision making. *Public Health Res Pract* 2017;27:e2731728.

17 UK National Screening Committee. Guidance for the development, production and review of information to support UK population screening programmes. 2018. https://www.gov.uk/government/publications/uk-national-screening-committee-information-development-guidance (accessed 17 Aug 2018).

18 European Commission. *European guidelines for quality assurance in cervical cancer screening. 2nd edition.* Luxembourg: : Office for Official Publications of the European Communities 2008. http://screening.iarc.fr/doc/ND7007117ENC_002.pdf (accessed 17 Aug 2018).

For peer review only

19 General Medical Council. Consent: patients and doctors making decisions together. 2008. https://www.gmc-uk.org/ethical-guidance/ethical-guidance-for-doctors/consent (accessed 17 Aug 2018).

20 Entwistle VA, Carter S, Trevena L, *et al.* Communicating about screening. *Br Med J* 2008;333:789–91.

21 Gigerenzer G, Gaissmaier W, Kurz-Milcke E, *et al.* Helping doctors and patients make sense of health statistics. *Psychol Sci Public Interest* 2007;8:53–96.

22 Fagerlin A, Zikmund-Fisher BJ, Ubel PA. Helping patients decide: ten steps to better risk communication. *J Natl Cancer Inst* 2011;103:1436–1443.

23 Schwartz LM, Woloshin S, Black WC, *et al.* The role of numeracy in understanding the benefit of screening mammography. *Ann Intern Med* 1997;127:966–972.

24 Spiegelhalter D. Risk and uncertainty communication. *Annu Rev Stat Its Appl* 2017;4:31–60.

25 Budescu DV, Por H-H, Broomell SB, *et al.* The interpretation of IPCC probabilistic statements around the world. *Nat Clim Change* 2014;4:508–512.

26 Knapp P, Raynor DK, Berry DC. Comparison of two methods of presenting risk information to patients about the side effects of medicines. *BMJ Qual Saf* 2004;13:176–180.

27 Knapp P, Gardner PH, Woolf E. Combined verbal and numerical expressions increase perceived risk of medicine side-effects: A randomized controlled trial of EMA recommendations. *Health Expect* 2016;19:264–274.

28 Peters E, Hart PS, Tusler M, *et al.* Numbers matter to informed patient choices: A randomized design across age and numeracy levels. *Med Decis Making* 2014;34:430–442.

For peer review only

29 Visschers VH, Meertens RM, Passchier WW, *et al.* Probability information in risk communication: A review of the research literature. *Risk Anal* 2009;29:267–287.

30 Young S, Oppenheimer DM. Effect of communication strategy on personal risk perception and treatment adherence intentions. *Psychol Health Med* 2009;14:430–442.

31 Zipkin DA, Umscheid CA, Keating NL, *et al.* Evidence-based risk communication: a systematic review. *Ann Intern Med* 2014;161:270–280.

32 Pighin S, Savadori L, Barilli E, *et al.* The 1-in-X effect on the subjective assessment of medical probabilities. *Med Decis Making* 2011;31:721–729.

33 Pighin S, Savadori L, Barilli E, *et al.* Communicating Down syndrome risk according to maternal age: “1-in-X” effect on perceived risk. *Prenat Diagn* 2015;35:777–82.

34 Sirota M, Juanchich M, Kostopoulou O, *et al.* Decisive evidence on a smaller-than-you-think phenomenon: revisiting the “1-in-X” effect on subjective medical probabilities. *Med Decis Making* 2014;34:419–429.

35 Sirota M, Juanchich M, Petrova D, *et al.* Health professionals prefer to communicate risk-related numerical information using “1-in-X” ratios. *Med Decis Making* 2018;38:366–376.

36 Sirota M, Juanchich M, Bonnefon J-F. 1-in-X" bias: “1-in-X” format causes overestimation of health-related risks. *J Exp Psychol Appl* Published Online First: 2018.

37 Trevena LJ, Zikmund-Fisher BJ, Edwards A, *et al.* Presenting quantitative information about decision outcomes: a risk communication primer for patient decision aid developers. *BMC Med Inform Decis Mak* 2013;13:S7.

For peer review only

38 Zikmund-Fisher BJ. Continued use of 1-in-X risk communications is a systemic problem. *Med Decis Making* 2014;34:412–413.

39 Akl EA, Oxman AD, Herrin J, *et al.* Using alternative statistical formats for presenting risks and risk reductions. *Cochrane Database Syst Rev* 2011;3:CD006776.

40 Covey J. A meta-analysis of the effects of presenting treatment benefits in different formats. *Med Decis Making* 2007;27:638–654.

41 Gigerenzer G, Edwards A. Simple tools for understanding risks: From innumeracy to insight. *Br Med J* 2003;327:741−744.

42 Galesic M, Garcia-Retamero R, Gigerenzer G. Using icon arrays to communicate medical risks: Overcoming low numeracy. *Health Psychol* 2009;28:210–216.

43 Garcia-Retamero R, Cokely ET. Designing visual aids that promote risk literacy: A systematic review of health research and evidence-based design heuristics. *Hum Factors* 2017;59:582–627.

44 Okan Y, Garcia-Retamero R, Cokely ET, *et al.* Individual differences in graph literacy: Overcoming denominator neglect in risk comprehension. *J Behav Decis Mak* 2012;25:390–401.

45 Paling J. Strategies to help patients understand risks. *Br Med J* 2003;327:745–748.

46 Schapira MM, Nattinger AB, McHorney CA. Frequency or probability? A qualitative study of risk communication formats used in health care. *Med Decis Making* 2001;21:459–467.

47 Jørgensen KJ, Gøtzsche PC. Presentation on websites of possible benefits and harms from screening for breast cancer: Cross sectional study. *Br Med J* 2004;328:148.

For peer review only

48 Rychetnik L, Carter SM, Abelson J, *et al.* Enhancing citizen engagement in cancer screening through deliberative democracy. *J Natl Cancer Inst* 2013;105:380–386.

49 Raffle AE. Information about screening–is it to achieve high uptake or to ensure informed choice? *Health Expect* 2001;4:92–98.

50 Attena F, Cancellieri M, Pelullo CP. Scarce information about breast cancer screening: An Italian websites analysis. *Medicine (Baltimore)* 2016;95:e5615.

51 Ghanouni A, Meisel SF, Hersch J, *et al.* Information on “overdiagnosis” in breast cancer screening on prominent United Kingdom-and Australia-oriented health websites. *PLoS One* 2016;11:e0152279.

52 Kolthoff SK, Hestbech MS, Jørgensen KJ, *et al.* Do invitations for cervical screening provide sufficient information to enable informed choice? A cross-sectional study of invitations for publicly funded cervical screening. *J R Soc Med* 2016;109:274–281.

53 Slaytor EK, Ward JE. How risks of breast cancer and benefits of screening are communicated to women: Analysis of 58 pamphlets. *Br Med J* 1998;317:263–264.

54 Jørgensen KJ, Gøtzsche PC. Content of invitations for publicly funded screening mammography. *Br Med J* 2006;332:538–541.

55 McHugh SM, Corrigan M, Morney N, *et al.* A quantitative assessment of changing trends in internet usage for cancer information. *World J Surg* 2011;35:253–257.

56 Liebl P, Seilacher E, Koester M-J, *et al.* What cancer patients find in the internet: The visibility of evidence-based patient information - Analysis of information on German websites. *Oncol Res Treat* 2015;38:212–218.

For peer review only

57 Phillips K, Hersch J, Turner R, *et al.* The influence of the 'cancer effect'on young women's responses to overdiagnosis in cervical screening. *Patient Educ Couns* 2016;99:1568–1575.

58 Brodersen J, Schwartz LM, Heneghan C, *et al.* Overdiagnosis: what it is and what it isn't. *BMJ Evid-Based Med* 2018;23:1–3.

59 Sasieni P, Castanon A, Cuzick J, *et al.* Effectiveness of cervical screening with age: population based case-control study of prospectively recorded data. *Br Med J* 2009;339:b2968.

60 Forbes LJ, Ramirez A-J, list) E group on I about BS. Offering informed choice about breast screening. *J Med Screen* 2014;21:194–200.

61 Dieng M, Trevena L, Turner RM, *et al.* What Australian women want and when they want it: Cervical screening testing preferences, decision-making styles and information needs. *Health Expect* 2013;16:177–188.

62 Waller J, Whitaker KL, Winstanley K, *et al.* A survey study of women's responses to information about overdiagnosis in breast cancer screening in Britain. *Br J Cancer* 2014;111:1831–1835.

63 Nagler RH, Franklin Fowler E, Gollust SE. Women's awareness of and responses to messages about breast cancer overdiagnosis and overtreatment. *Med Care* 2017;55:879–885.

64 Ghanouni A, Renzi C, Waller J. Improving public understanding of 'overdiagnosis' in England: A population survey assessing familiarity with possible terms for labelling the concept and perceptions of appropriate terminology. *BMJ Open* 2018;8:e021260.

65 Gigerenzer G. Full disclosure about cancer screening. *Br Med J* 2016;352:h6967.

For peer review only

66 Prasad V, Lenzer J, Newman DH. Why cancer screening has never been shown to “save lives”—and what we can do about it. *Br Med J* 2016;352:h6080.

67 Natter HM, Berry DC. Effects of presenting the baseline risk when communicating absolute and relative risk reductions. *Psychol Health Med* 2005;10:326–334.

68 Sorensen L, Gyrd-Hansen D, Kristiansen IS, *et al.* Laypersons’ understanding of relative risk reductions: randomised cross-sectional study. *BMC Med Inform Decis Mak* 2008;8:31.

69 Covey J. The effects of absolute risks, relative risks, frequencies, and probabilities on decision quality. *J Health Commun* 2011;16:788–801.

70 Bodemer N, Meder B, Gigerenzer G. Communicating relative risk changes with baseline risk: Presentation format and numeracy matter. *Med Decis Making* 2014;34:615–626.

71 Jenkins SC, Harris AJ, Lark RM. When unlikely outcomes occur: the role of communication format in maintaining communicator credibility. *J Risk Res* 2018.

72 Gurmankin AD, Baron J, Armstrong K. The effect of numerical statements of risk on trust and comfort with hypothetical physician risk communication. *Med Decis Making* 2004;24:265–271.

73 McDowell M, Rebitschek FG, Gigerenzer G, *et al.* A simple tool for communicating the benefits and harms of health interventions: A guide for creating a Fact Box. *MDM Policy Pract* 2016;1:1–10.

74 McDowell M, Gigerenzer G, Wegwarth O, *et al.* Effect of tabular and icon fact box formats on comprehension of benefits and harms of prostate cancer screening: a randomized trial. *Med Decis Making* 2019;39:41–56.

For peer review only

75 Woloshin S, Schwartz LM, Black WC, *et al.* Cancer Screening campaigns — getting past uninformative persuasion. *N Engl J Med* 2012;367:1677–1679.

76 Schwartz LM, Woloshin S, Floyd J. Fowler J, *et al.* Enthusiasm for Cancer Screening in the United States. *JAMA* 2004;291:71–8.

77 Waller J, Osborne K, Wardle J. Enthusiasm for cancer screening in Great Britain: a general population survey. *Br J Cancer* 2015;112:562–566.

78 Nagler RH. Adverse outcomes associated with media exposure to contradictory nutrition messages. *J Health Commun* 2014;19:24–40.

79 Han PK, Kobrin SC, Klein WM, *et al.* Perceived ambiguity about screening mammography recommendations: association with future mammography uptake and perceptions. *Cancer Epidemiol Prev Biomark* 2007;16:458–466.

80 Cheng C, Dunn M. How well are health information websites displayed on mobile phones? Implications for the readability of health information. *Health Promot J Austr* 2017;28:15–20.

81 Longman T, Turner RM, King M, *et al.* The effects of communicating uncertainty in quantitative health risk estimates. *Patient Educ Couns* 2012;89:252–259.

82 Sladakovic J, Jansen J, Hersch J, *et al.* The differential effects of presenting uncertainty around benefits and harms on treatment decision making. *Patient Educ Couns* 2016;99:974–980.

83 Han PK. Conceptual, methodological, and ethical problems in communicating uncertainty in clinical evidence. *Med Care Res Rev* 2013;70:14S–36S.

84 Ghanouni A, Renzi C, Meisel SF, *et al.* Common methods of measuring ‘informed choice’in screening participation: Challenges and future directions. *Prev Med Rep* 2016;4:601–607.

For peer review only

85 Peters E, Dieckmann N, Dixon A, *et al.* Less is more in presenting quality information to consumers. *Med Care Res Rev* 2007;64:169–190.

86 Waller J, Douglas E, Whitaker KL, *et al.* Women's responses to information about overdiagnosis in the UK breast cancer screening programme: a qualitative study. *BMJ Open* 2013;3:e002703.

87 Hestbech MS, Gyrd-Hansen D, Kragstrup J, *et al.* Effects of numerical information on intention to participate in cervical screening among women offered HPV vaccination: A randomised study. *Scand J Prim Health Care* 2016;34:401–419.

88 Bunge M, Mühlhauser I, Steckelberg A. What constitutes evidence-based patient information? Overview of discussed criteria. *Patient Educ Couns* 2009;78:316–328.

For peer review only

Figure captions

Figure 1. Flowchart representing the website selection process.

Figure 2. Results of analysis of content and quantitative presentation formats for information about (a) cervical screening benefits, (b) risks, (c) possible results, and (d) cervical cancer statistics. A total of 14 websites were analyzed. Items marked with an asterisk were added by the authors to the checklist by Kolthoff et al.[52]. Details concerning instances in which multiple formats were used for a given information item appear in Supplementary materials (Table S3).

For peer review only

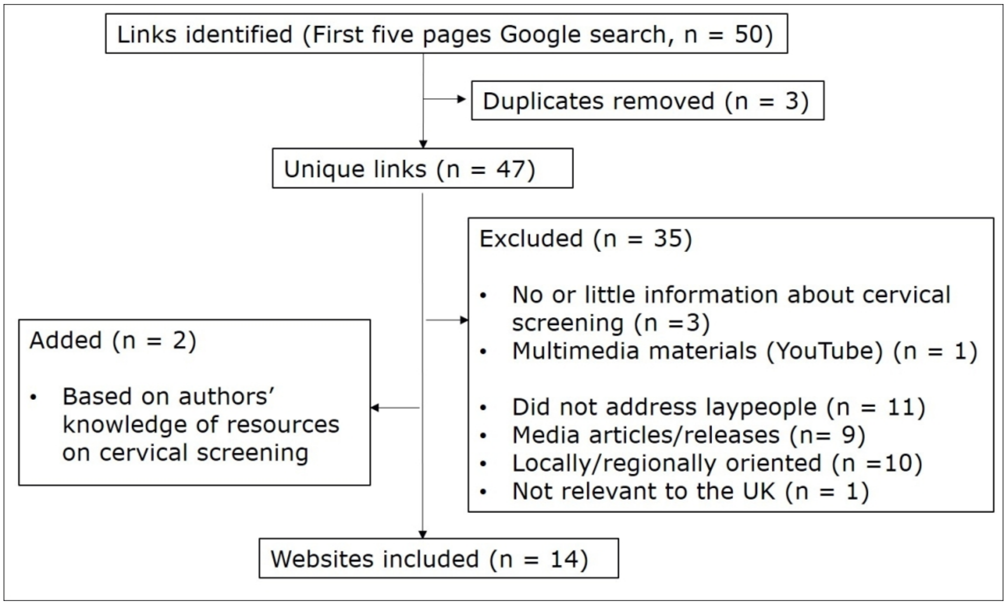

Figure 1. Flowchart representing the results of the website selection after applying exclusion criteria.

241x144mm (150 x 150 DPI)

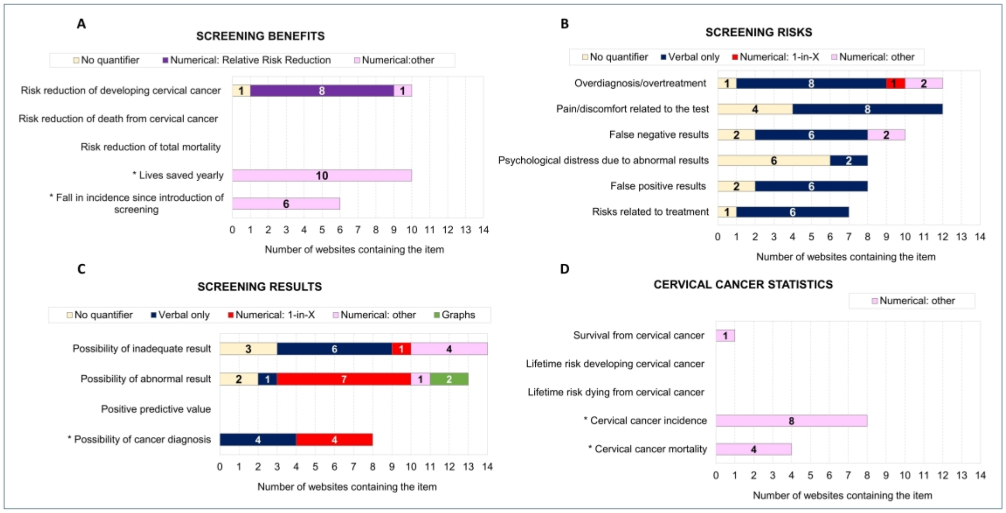

Figure 2. Results of analysis of content and quantitative presentation formats for information about (a) cervical screening benefits, (b) risks, (c) possible results, and (d) cervical cancer statistics. A total of 14 websites were analyzed. Items marked with an asterisk were added by the authors to the checklist by Kolthoff et al.[30]. Details concerning instances in which multiple formats were used for a given information item appear in Supplementary materials (Table S3).

1722x872mm (96 x 96 DPI)

Supplementary materials for BMJ Open

How is cervical screening communicated in UK websites? A cross-sectional analysis of content and quantitative presentation formats

Yasmina Okan,[1] * Samuel G. Smith,[2] Wändi Bruine de Bruin,[1,3]

[1]Centre for Decision Research, Leeds University Business School, University of Leeds, Leeds, UK

[2] Leeds Institute of Health Sciences, University of Leeds, Leeds, UK

[3]Department of Engineering and Public Policy, Carnegie Mellon University, Pittsburgh, US

For peer review only

* Corresponding author at Centre for Decision Research, Leeds University Business School, University of Leeds, Charles Thackrah Building, Leeds, LS2 9LB, UK; E-mail: y.okan@leeds.ac.uk, Phone: +44 113 343 2622.

Table S1. Websites coded, date of access for analysis, and estimated visits.

Organization	Link	Date	Total monthly visits (million) [c]	% of UK visitors [c]
British Society for Colposcopy and Cervical Pathology	https://www.bsccp.org.uk/	28/04/2017	- [d]	- [d]
BootsWebMD [a]	https://www.webmd.boots.com/	09/01/2018	- [d]	- [d]
Bupa UK	https:/www.bupa.co.uk	27/04/2017	1.11	86.15%
Cancer Research UK	https://www.cancerresearchuk.org	27/04/2017	4.46	47.45%
Jo's Cervical Cancer Trust [a]	https://www.jostrust.org.uk	27/04/2017	0.21	68.64%
LGBT Foundation [b]	http://lgbt.foundation	27/04/2017	0.06	66.66%
MacMillan Cancer Support [a]	https://www.macmillan.org.uk	27/04/2017	1.13	65.03%
Marie Stopes UK [b]	https://www.mariestopes.org.uk	27/04/2017	0.06	46.77%
NHS Choices (England) [a]	http://www.nhs.uk	20/03/2017	33.53	71.60%
NHS Inform (Scotland) [a]	https://www.nhsinform.scot	20/03/2017	2.96	55.43%
Patient Info	https://patient.info	27/04/2017	4.06	47.05%
Public Health Agency Northern Ireland [a]	http://www.cancerscreening.hscni.net	26/04/2017	0.40 [e]	81.37% [e]
Public Health Wales [a]	http://www.cervicalscreeningwales.wales.nhs.uk	20/04/2017	1.07 [e]	87.68% [e]
Women's Health Concern	https://www.womens-health-concern.org	28/04/2017	0.12	19.11%

Note: [a] Electronic leaflets and/or factsheets for the general public were available to download via a link this website, and were thus considered part of this resource; [b] Only an electronic leaflet was available and thus the analysis focuses on this resource; [c] Estimated data obtained from SimilarWeb (www.similarweb.com), representing visits in June 2019. Estimates aggregate data across all website subdomains and are computed by extrapolation from a small panel of users, and therefore need to be interpreted with caution; [d] Estimates not available due to insufficient data; [e] Estimates based on the general domain ("hscni.net" and "wales.nhs.uk")

For peer review only

Table S2. Codes from Kolthoff et al.'s (2016) checklist, with examples of corresponding statements. Codes marked with an asterisk were added by the authors.

Information item	Example statements
**Screening benefits**	
Risk reduction of developing cervical cancer	Regular screening can cut the risk of getting cervical cancer by 75%.
	Cervical screening prevents 8 out of 10 cervical cancers from developing.
Risk reduction of death from cervical cancer	No statements found
Risk reduction of total mortality	No statements found
* Lives saved yearly	Cervical screening saves around 5,000 lives every year in the UK.
	Over 1000 lives are saved every year through regular screening.
* Fall in incidence since introduction of screening	The number of women who develop cervical cancer has halved since the 1980s due to most women regularly having cervical screening.
	Since the screening programme was introduced in the 1980s, the number of cervical cancer cases has decreased by about 7% each year.
**Screening risks**	
Overdiagnosis/ overtreatment	The test can pick up minor abnormalities in cervical cells which could clear up by themselves.
	Your screening test may show up mild cell changes in your cervix, which would never have gone on to become cancer or caused you any problems. If this happens, you may receive treatment that you don't really need.
Pain/discomfort related to the test	Cervical screening tests are not painful, although some women find the speculum uncomfortable.
	You might feel some discomfort or pain - try to relax by taking slow, deep breaths as it may hurt more if you are tense.
False negative results	No screening test is 100% accurate. Some tests will be falsely reassuring (so-called false negative results) - where the test is reported as normal but an abnormality is present.
	Regular screening detects up to 80% of cervical abnormalities.
Psychological distress due to abnormal results	A diagnosis of and treatment for cervical abnormalities can cause significant anxiety for many women.
	Many women worry when an abnormality is found.

For peer review only

False positive results	False positive results might wrongly show changes in your cervix.
	Very occasionally, screening can show you have abnormal cells when in fact there's no problem. This is called a false positive result.
Risks related to treatment	For a few women, the treatments may cause problems such as bleeding afterwards or a small increase in future pregnancies of having the baby early.
	Very rarely, the cervix can become tightly closed after treatment. This known as stenosis. It can make it harder for the sperm to enter the womb and so can affect your chances of becoming pregnant naturally.
**Screening results**	
Possibility of inadequate result	Sometimes test results are declared 'unsatisfactory' because the slide was poorly prepared or difficult to read. In such cases, the test needs to be repeated and the patient will be called back.
	An inadequate result does not mean your smear was abnormal but that it was unreadable. This means that there were not enough cells in the sample and happens in about 3 out of every 100 tests taken.
Possibility of abnormal result	Most abnormal results from screening tests show only very minor changes.
	About 1 in 20 women will have a smear test result which is abnormal.
Positive predictive value	No statements found
* Possibility of cancer diagnosis	A cervical screening test can very occasionally find early cervical cancer. Most women with an abnormal test result have early cell changes and not cancer.
	It is extremely rare for cancer to be diagnosed from a cervical screening test. Less than one in 1,000 women's test results show invasive cancer.
**Cervical cancer statistics**	
Survival from cervical cancer	Overall, almost 2 out of 3 women (63%) survive ten years or more, and more than 8 out of 10 women (83%) will live at least one year.
Lifetime risk of developing cervical cancer	No statements found
Lifetime risk of dying from cervical cancer	No statements found
* Cervical cancer incidence	Around 3,000 women are diagnosed with cancer of the cervix every year.
	Each year in Northern Ireland, about 103 women are diagnosed with cervical cancer.
* Cervical cancer mortality	Sadly around 900 women die of cervical cancer each year in England.
	In Northern Ireland, 20–30 women die each year from cervical cancer.

For peer review only

Table S3a. Information items about cervical screening benefits, risks, results, and cervical cancer statistics (Part 1)

	BSCCP	Boots WebMD	Bupa UK	Cancer Research UK	Jo's Cervical Cancer Trust	LGBT Foundation	MacMillan Cancer Support
**Screening benefits**							
Risk reduction of developing cervical cancer	-	-	-	RR	RR	RR	0
Risk reduction of death from cervical cancer	-	-	-	-	-	-	-
Risk reduction of total mortality	-	-	-	-	-	-	-
* Lives saved yearly	-	-	N	N	N	-	N
* Fall in incidence since the introduction of screening [a]	N	-	-	N	-	-	N
**Screening risks**							
Overdiagnosis/overtreatment	V	-	V	V	V, N	V	V
Pain/discomfort related to the test	-	0	0	0	V	V	V
False negative results	-	-	0	0	V, N	-	V
Psychological distress due to abnormal results	0	-	0	0	V	V	0
False positive results	V	-	V	V	-	-	V
Risks related to treatment	V	-	-	V	V	-	V
**Screening results**							
Possibility of inadequate result	0	V	V, N	V	V	0	V
Possibility of abnormal result	1X, N	0	1X	1X, N	V, 1X	V, 1X	V
Positive predictive value	-	-	-	-	-	-	-
* Possibility of cancer diagnosis	V, 1X, N	-	-	V	V, 1X	V	V
**Cervical cancer statistics**							
Survival from cervical cancer	-	N	-	-	-	-	-
Lifetime risk of developing cervical cancer	-	-	-	-	-	-	-
Lifetime risk of dying from cervical cancer	-	-	-	-	-	-	-
* Cervical cancer incidence	-	N	N	-	N	-	-
* Cervical cancer mortality	-	-	-	-	N	N	-
**Type of appeals**							
Participation	x	x	x	x	x	x	x
Informed decision making	-	-	x	-	x	-	-

Note: - = item not present; 0 = not quantified; V = verbal quantifier; RR = numerical relative risk reduction; 1X = numerical 1-in-X; N = numerical (other); G = graph; x = item present (coding format not applicable); * codes added by the authors; [a] includes estimates of incidence at different time points, as well as any mention to the magnitude of the fall in incidence (e.g., rates have halved).

For peer review only

Table S3b. Information items about cervical screening benefits, risks, results, and cervical cancer statistics (Part 2)

	Marie Stopes UK	NHS Choices (England)	NHS Inform (Scotland)	Patient Info	PHA Northern Ireland	Public Health Wales	Women's Health Concern
**Screening benefits**							
Risk reduction of developing cervical cancer	RR	N	RR	RR	RR	RR	-
Risk reduction of death from cervical cancer	-	-	-	-	-	-	-
Risk reduction of total mortality	-	-	-	-	-	-	-
* Lives saved yearly	N	N	N	N	-	N	N
* Fall in incidence since the introduction of screening [a]	-	N	-	N	-	N	-
**Screening risks**							
Overdiagnosis/overtreatment	0	V	V	V, N	-	V, 1X	V
Pain/discomfort related to the test	V	V	-	V	V	V	0
False negative results	-	V	V	V	V	V	V, N
Psychological distress due to abnormal results	-	0	-	0	-	-	-
False positive results	-	V	0	0	-	V	-
Risks related to treatment	-	V	-	-	0	V	-
**Screening results**							
Possibility of inadequate result	0	V, N	V	N	V, N	1X, N	V
Possibility of abnormal result	0	V, 1X, N, G	V, 1X, G	V, N	V, 1X, N	V, 1X, N	-
Positive predictive value	-	-	-	-	-	-	-
* Possibility of cancer diagnosis	-	V, 1X	-	V, 1X	-	V	-
**Cervical cancer statistics**							
Survival from cervical cancer	-	-	-	-	-	-	-
Lifetime risk of developing cervical cancer	-	-	-	-	-	-	-
Lifetime risk of dying from cervical cancer	-	-	-	-	-	-	-
* Cervical cancer incidence	N	N	-	N	N	N	-
* Cervical cancer mortality	-	-	-	-	N	N	-
**Type of appeals**							
Participation	-	-	x	x	x	x	x
Informed decision making	-	x	x	-	-	x	x

Note: - = item not present; 0 = not quantified; V = verbal quantifier; RR = numerical relative risk reduction; 1X = numerical 1-in-X; N = numerical (other); G = graph; x = item present (coding format not applicable); * codes added by the authors; [a] includes estimates of incidence at different time points, as well as any mention to the magnitude of the fall in incidence (e.g., rates have halved).

For peer review only
